# Archaeal GPN-loop GTPases involve a lock-switch-rock mechanism for GTP hydrolysis

**DOI:** 10.1128/mbio.00859-23

**Published:** 2023-11-14

**Authors:** Lukas Korf, Xing Ye, Marian S. Vogt, Wieland Steinchen, Mohamed Watad, Chris van der Does, Maxime Tourte, Shamphavi Sivabalasarma, Sonja-Verena Albers, Lars-Oliver Essen

**Affiliations:** 1 Department of Chemistry, Philipps University, Marburg, Germany; 2 University of Freiburg, Institute of Biology, Molecular Biology of Archaea, Freiburg, Germany; 3 Center for Synthetic Microbiology (SYNMIKRO), Karl-von-Frisch-Strasse, Marburg, Germany; 4 Spemann Graduate School of Biology and Medicine, University of Freiburg, Freiburg, Germany; University of California Irvine, Irvine, California, USA; University of California Irvine, Irvine, California, USA

**Keywords:** GPN-loop, GTPase, sulfolobus, RNA polymerase II, allostery

## Abstract

Three GPN-loop GTPases, GPN1–GPN3, are central to the maturation and trafficking of eukaryotic RNA polymerase II. This GTPase family is widely represented in archaea but typically occurs as single paralogs. Structural analysis of the GTP- and GDP-bound states of the *Sulfolobus acidocaldarius* GPN enzyme (*Sa*GPN) showed that this central GPN-loop GTPase adopts two distinct quaternary structures. In the GTP-bound form, the γ-phosphate induces a closed dimeric arrangement by interacting with the GPN region that is relaxed upon hydrolysis to GDP. Consequently, a rocking-like motion of the two protomers causes a major allosteric structural change toward the roof-like helices. Using a lock-switch-rock mechanism, homo- and heterodimeric GPN-like GTPases are locked in the GTP-bound state and undergo large conformational changes upon GTP hydrolysis. A Δ*saGPN* strain of *S. acidocaldarius* was characterized by impaired motility and major changes in the proteome underscoring its functional relevance for *S. acidocaldarius in vivo*.

**IMPORTANCE:**

GPN-loop GTPases have been found to be crucial for eukaryotic RNA polymerase II assembly and nuclear trafficking. Despite their ubiquitous occurrence in eukaryotes and archaea, the mechanism by which these GTPases mediate their function is unknown. Our study on an archaeal representative from *Sulfolobus acidocaldarius* showed that these dimeric GTPases undergo large-scale conformational changes upon GTP hydrolysis, which can be summarized as a lock-switch-rock mechanism. The observed requirement of *Sa*GPN for motility appears to be due to its large footprint on the archaeal proteome.

## INTRODUCTION

GTPases are a large family of GTP-binding and -hydrolyzing enzymes that are widely distributed across all three domains of life (1). They contain a highly conserved GTPase domain (G domain) housing five fingerprint motifs responsible for coordination of GTP and catalysis: G1 (also P-loop or Walker A motif) interacts with the 5′ phosphate moieties of GTP, the G2 and G3 motifs are required for coordination of a magnesium ion essential for catalysis, the latter of which specifically accommodates the 5’ γ-phosphate of GTP, and G4 and G5 establish specific binding of the nucleotides’ guanine base (2, 3). As GTPases cycle between their GTP and GDP-bound states via their intrinsic GTP hydrolytic activity, they often function as molecular switches differentially regulating a plethora of downstream effector proteins involved in crucial cellular processes (2, 4, 5).

Most GTPases belong either to the TRAFAC (translation factor association) class involved in translation, signal transduction and intracellular transport, or the SIMIBI (signal recognition particle, MinD, and BioD) class; members of the latter engage in protein localization and trafficking, membrane transport, and chromosome partitioning (1, 4, 6). Many well-studied GTPases, such as Ras and heterotrimeric G proteins (7–9), belong to the TRAFAC class, whose members generally do not form nucleotide-dependent dimers (1). In contrast, homo- and heterodimerization of SIMIBI class proteins like the signal recognition particle (SRP) and SRP receptor GTPases depends on ATP- or GTP-binding (4, 10) and is hence relevant to regulation of their intrinsic ATP/GTP hydrolysis activity, specific interactions with effector proteins, and their biological functions (6, 11, 12).

A novel type of SIMIBI GTPase, the GPN-loop GTPase, was discovered by structural analysis of the GPN-loop GTPase PAB0955 (*Pa*GPN, Uniprot: Q9UYR9) from the euryarchaeon *Pyrococcus abyssi* (13). *Pa*GPN adopts a homodimeric topology, whose quaternary assembly was found to be independent of GTP binding (13). This characteristic of GPN-loop GTPases sets them aside from other SIMIBI GTPases, which require switch-dependent changes of their quaternary state to fulfill their biological function (6). Another feature is the eponymous GPN motif in the G domain that is highly conserved in archaeal *Pa*GPN and eukaryotic orthologs. This motif (Gly-Pro-Asn) is inserted in between the SIMIBI class motifs G2 and G3. GPN-loop GTPases were described to occur only in archaea and eukaryotes, but not in bacteria (14). Eukaryotes typically feature three GPN-loop GTPase paralogs: GPN1 (annotated before as Npa3, XAB1, or MBD*in*), GPN2 and GPN3. These paralogs play essential roles in nuclear localization and biogenesis of the RNA polymerase II (14–17). In yeast, deletion of genes encoding the XAB1 homologs GPN1, GPN2 or GPN3 was found to be lethal (18). Furthermore, yeast GPNs assemble into heterodimers with different yGPN combinations, which appear to be irrespective of the nucleotide-bound state of the GPNs as pull-down assays, FRET, and molecular modeling studies indicate (14, 19).

The biological function of archaeal GPN-loop GTPases remains unknown, although they exhibit the highest sequence similarities with eukaryotic GPN1 orthologs (1, 19). Nevertheless, the crystal structures of *P. abyssi* GPN (PDB: 1YR6 [apo form], 1YRA [GDP complex], 1YR7 [GTP-γ-S complex], 1YR8 [GTP complex], 1YR9 [GDP+HPO_4_^2−^ complex], 1YRB [GDP+Mg^2+^ complex], 2OXR [GDP+Mg^2+^ complex, after GTP hydrolysis]) and yeast GPN1 (also called Npa3, PDB: 5HCI [GDP complex], 5HCN [GMPPCP complex]) revealed a similar homodimeric topology, nucleotide coordination, and a mode of catalysis, which involves the asparagine residue of the GPN motif (13, 16).

For long time, the cellular function of GPN-loop GTPases remained enigmatic. *Homo sapiens* GPN1 was described first as an XPA-binding protein 1 (XAB1) or MBD2-interacting protein (MBD*in*) because of its interaction with the nucleotide excision repair protein Xeroderma pigmentosum group A protein (XPA) and methyl-CpG-binding protein MBD2, thus putting forward a role in DNA repair and methylation-dependent transcription (20, 21). Elucidation of the protein interaction network of all three GPN proteins in yeast revealed their essential role in the regulation of nuclear import of multiple RNA polymerase II (RNAPII) subunits (22, 23). The interaction of yeast GPN1/Npa3 with RNAPII subunit Rpb1 *in vitro* was stronger in the presence of GTP than GDP (24). Furthermore, mutations in the G1, G2, G3, and GPN motifs of GPN1/Npa3 were either lethal or resulted in a slow growth phenotype in yeast (23), collectively suggesting a link between the nucleotide-bound state of GPN GTPases and their cellular function.

In the archaeon *Sulfolobus acidocaldarius*, the GTPase *Sa*ci1281 (from hereon: *Sa*GPN) represents the solitary GPN-loop GTPase. We previously identified *Sa*GPN as an interaction partner of the Ser/Thr specific protein phosphatase PP2A (25), which we anticipate to play an important role in intercellular signaling pathways because *S. acidocaldarius* possesses a diverse phosphoproteome (26). Interestingly, PP2A interacts with ArnA and ArnB, which are described as negative regulators of the assembly of the archaellum (25, 27, 28). PP2A deletion strains are therefore hypermotile and defective in cell size regulation and metabolism (26).

We investigated the *in vivo* function of *Sa*GPN in *S. acidocaldarius*, focusing on its potential role in the regulation of archaella synthesis. We also analyzed the protein structure and conformational changes of *Sa*GPN by crystallization and HDX analysis. The results from this study shed light on the *in vivo* function of GPN-loop GTPase in archaea, and we will also present a novel activation mechanism of GPN-loop GTPases.

## RESULTS

### GPNs occur in all domains of life

**Fig 1:**
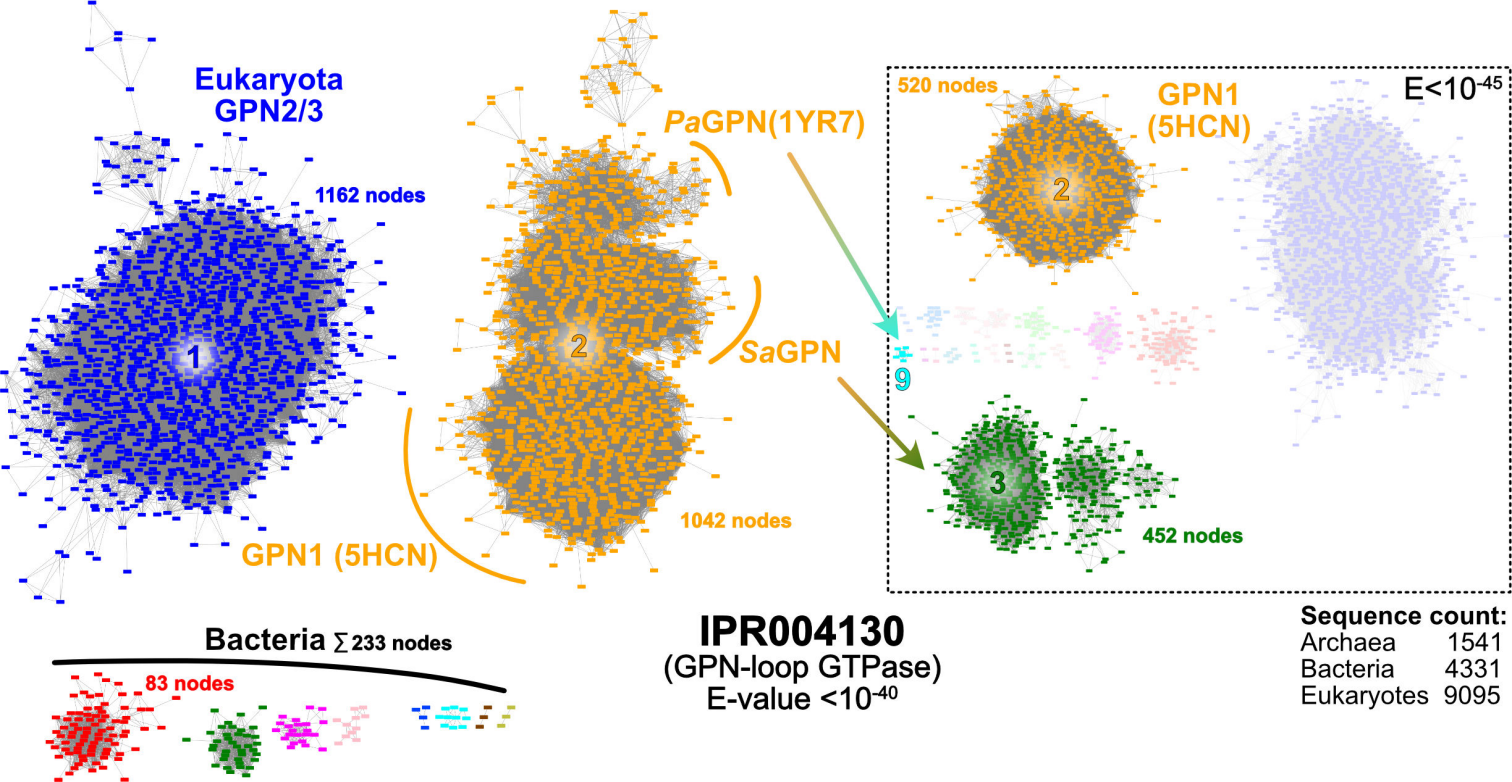
Sequence similarity network (SSN) of InterPro protein familiy IPR004130 (GPN-loop GTPases). The SSN shows connection between related proteins of the input protein familiy at the given *E*-value cutoff of 10^−40^. Cluster analysis numbers clusters based on member count, giving insights in overall cluster size. While the archaeal GPNs are clustered with GPN1 in cluster 2, other eukaryotic GPNs (GPN2/3) are located in cluster 1, revealing differences between GPN1 and its sister proteins. Additionally, cluster separation of cluster 2 is observable, where PaGPN, SaGPN, and GPN1 start to form their own subclusters. This is even more pronounced at a higher stringency of 10^−45^ (dashed box), where SaGPN separates from cluster 2 alongside most other archaeal GPNs into cluster 3 (452 nodes) and PaGPN is clustered away into its own cluster 9 (cyan). Nodes not found in clusters 2 and 3 clustered in smaller/singleton subclusters, which are not displayed for clarity. Notably, bacterial members of IPR004130 (lower left) miss the GPN-loop motif and are assignable to ARF-like GTPases (Fig. S10).

To provide an overview of GPN-loop GTPases (IPR004130), we analyzed and visualized this protein family using a sequence similarity network (SSN), which provides insights into protein relationships based on different sequence alignment scores (Fig. 1). Thereby, nodes correspond to protein sequences, and each edge represents one relation, which scales in stringency with decreasing *E*-values, allowing clustering based on this alignment threshold (29). Cluster analysis of IPR004130 with an *E*-value of 10^−40^ reveals that GPN-loop GTPases (GPNs) from archaea share a closer relation to GPN1 than to GPN2 and GPN3, which is consistent with the essential diversity of GPNs in eukaryotes (15). Moreover, GPN1 does not cluster with its sister paralogs GPN2/3, although the latter shares the same cluster, suggesting a greater functional discrepancy between GPN1 and GPN2/3 than between GPN2 and GPN3. Additionally, it appears that GPNs of TACK archaea, which include *S. acidocaldarius*, have a closer relationship with GPN1 than with other archaeal GPN-loop GTPases such as *P. abyssi* (Fig. 1). Furthermore, *Pyrococcus* and *Sulfolobus* GPNs begin to separate from each other at alignment scores with *E*-values of <10^−40^. Segregation became even more evident at a slightly increased *E*-value cutoff of <10^−45^, with the eukaryotic GPN1 clade being completely dissociated from archaeal GPNs. Likewise, *Pyrococcus*-like GPNs are almost completely clustered separate from the *Sa*GPN-loop GTPases (Fig. 1). This indicates, that despite sharing identity to some extent, these archaeal GTPases have some differences that are yet to be clarified. In our analysis of 1,541 unique archaeal sequences, belonging to 469 different organisms, we found that GPN-loop GTPases in archaea mostly occurred as singlets, with a total of 412 organisms. However, 57 archaeal organisms had two or more GPN paralogs, refuting the established assumption that archaea generally harbor only a single ortholog of GPN-loop GTPases (16, 19, 30).

Interestingly, 4,331 bacterial sequences are found in the SSN of IPR004130, which can be assigned to 1,859 different bacterial strains. Here, singlets also form the largest group in the bacterial domain (762 organisms); 410 organisms were identified with two paralogs, and 386 with three. In addition, some bacterial species even appear to have four or more paralogs, exceeding the number of three, GPN1–GPN3, as known in eukaryotes. However, all these bacterial members of IPR004130 share few relationships with the clusters dominated by either archaeal or eukaryotic members. Structure prediction and sequence alignments actually show that these bacterial GTPases miss the GPN motif and are structurally more related to ARF-type small GTPases (Fig. S10). This indicates that these bacterial members have been falsely annotated as GPN-loop GTPases and hence play a different and maybe more diverse role in bacteria when compared to eukaryotes or archaea. Furthermore, at an *E*-value of 10^−40^ archaeal orthologs assemble almost exclusively within a single, large cluster and thereby share links with almost every other archaeal GPN, whereas bacteria split into several small clusters that have only minor relationships.

### Biological effect of *saGPN* deletion on the archaeon *S. acidocaldarius*

**Fig 2:**
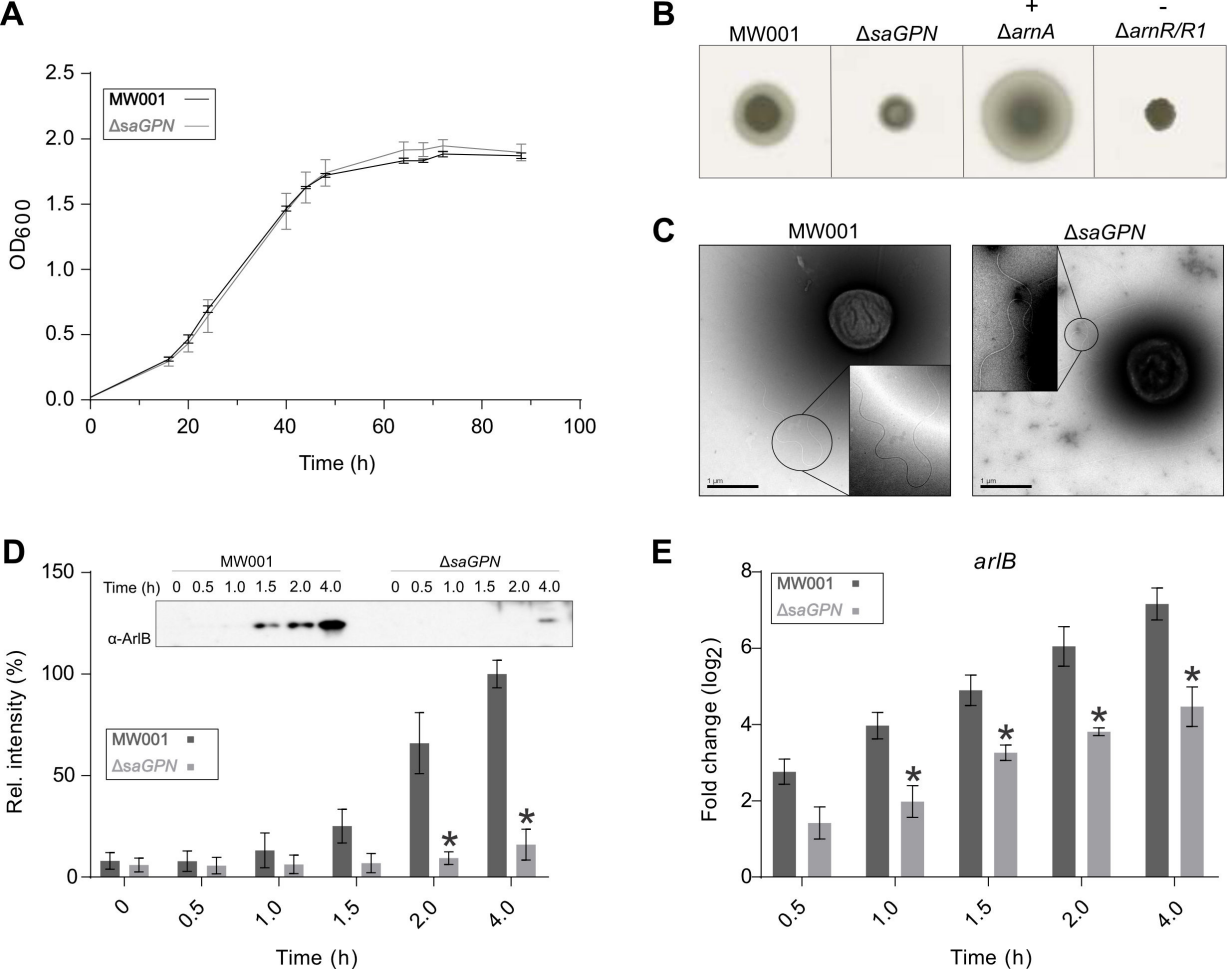
Deletion of saGPN impedes motility and arlB (flaB) expression in *S. acidocaldarius*. (**A**) Growth curves of *S. acidocaldarius* MW001 (black line) and Δ*saGPN* mutant (gray line) in nutrient-rich medium. (**B**) Motility assays. Same amounts of cells from tested *S. acidocaldarius* strains were spotted on semi-solid gelrite plates and incubated at 75°C for 5 days, then the plates were scanned and recorded. Δ*arnA* and Δ*arnR*/*R1* strains were used as hyper-motile and non-motile control, respectively. (**C**) Analysis of the archaellum formation in *S. acidocaldarius* strains. *S*. *acidocaldarius* MW001 and Δ*saGPN* knockout were cultivated in nutrient-depleted medium. After 4 h growth, cell samples were collected and applied to EM analysis. (**D**) ArlB expression on protein level and (**E**) *arlB* transcription level in *S. acidocaldarius*. *S. acidocaldarius* MW001 and Δ*saGPN* mutant were cultivated in nutrient-depleted medium for 4 h. Cell samples were taken at different time points, and analyzed by Western blotting with α-ArlB (left) and qRT-PCR (right). A representative Western blot is shown (**D**) and Western blots from biological triplicates were quantified. Relative transcription levels of arlB were normalized to secY. The values represent fold changes compared with the control from biological triplicates. Significant differences between MW001 (dark boxes) and Δ*saGPN* mutant (light boxes; *P* value < 0.05) were indicated by an asterisk.

*Sa*GPN (Saci_1281, Uniprot: Q4J9A7) was identified as a specific interactor of the serine/threonine protein phosphatase PP2A (25). In *S. acidocaldarius,* PP2A regulates growth, cell size, and swimming motility (26). To investigate the physiological role of *Sa*GPN in *S. acidocaldarius*, a markerless in-frame deletion mutant was constructed. Deletion of *saGPN* did not affect cell growth (Fig. 2A), whereas a pronounced decrease in swimming motility on semi-solid gelrite plates was observed (Fig. 2B). Interestingly, electron microscopy (EM) analysis showed that Δ*saGPN* cells still assembled archaella. However, these samples have to be taken after 4 h of starvation as otherwise archaella cannot be observed even in the wild type (WT) (Fig. 2C). To test whether deletion of *saGPN* affected *arlB* expression (encoding the archaellum filament protein, archaellin), Δ*saGPN* cells were starved for nutrients for 0–4 h, and *arlB* expression was analyzed by Western blot analysis and qRT-PCR (Fig. 2D). *arlB* expression generally increased during nutrient starvation in both the wild-type strain MW001 and Δ*saGPN* mutant. However, ArlB protein levels were significantly reduced in the Δ*saGPN* mutant compared with those in MW001. Similarly, decreased *arlB* expression was observed on the RNA level when comparing the Δ*saGPN* mutant with MW001 at 1, 1.5, 2, and 4 h after starvation (Fig. 2E). Thus, *Sa*GPN affects the archaellum regulation network and apparently acts as a positive regulator *in S. acidocaldarius*.

**Fig 3:**
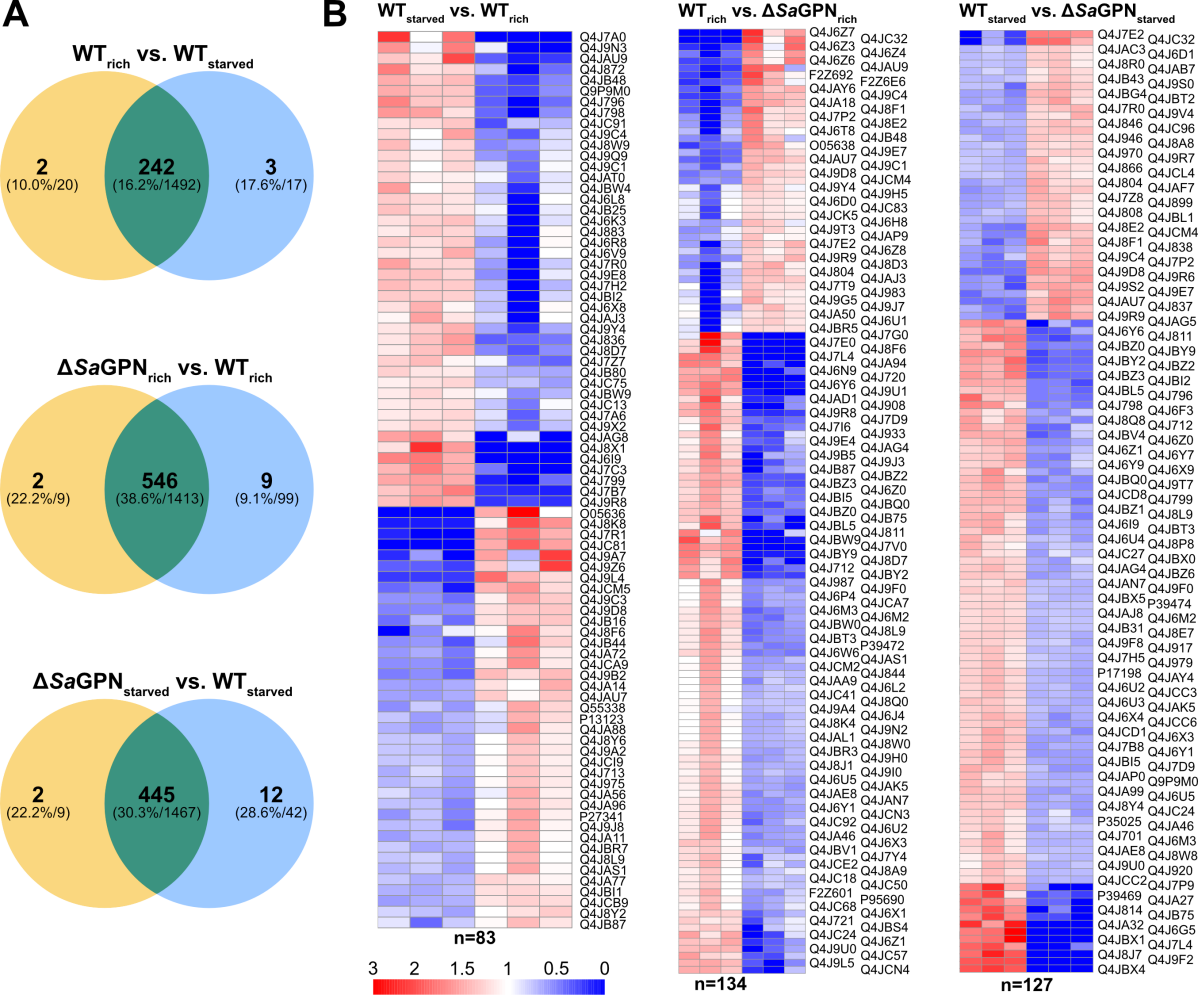
Knockout of *saGPN* has extensive ramifications on the *S. acidocaldarius* proteome. (**A**) Venn diagrams of proteomic data comparing different nutritional states of the wild type (WT) and *saGPN* deletion mutant. Diagrams show the number of overlapping and non-overlapping proteins that passed a one-paired *t*-test ≤0.01, to check for variation of the biological triplicates and the technical duplicates of their measurement, in bold. The proportion of these proteins filtered by the *t*-test to the total overlapping and non-overlapping proteins found is given in parentheses. The amounts of total proteins as identified by timsTOF, i.e. without *t*-test filtering, are given in absolute numbers next to them. (**B**) Heatmaps of proteins that exhibited highly affected expression levels (>50%) in their respective comparisons. The number of proteins that were severely affected is indicated below the maps. The scale bar represents the fold change of over-regulation (red) to under-regulation (blue). Uniprot identifiers of the respective proteins are indicated next to the rows.

To explore whether *Sa*GPN affects other functions in *S. acidocaldarius*, we performed a proteome-scale analysis of wild-type and Δ*saGPN S. acidocaldarius* strains. These were grown as biological triplicates and samples were taken from nutrient-rich and nutrient-starved conditions. Each sample was measured as technical duplicates using a timsTOF (trapped ion mobility spectrometry, time of flight) mass spectrometer before averaging and further downstream processing. Measurement of these proteomes led to the identification of 1,422–1,512 proteins per sample, which corresponded to a total of 1,627 different proteins of the 2,222 proteins encoded in the *S. acidocaldarius* genome. Comparing the two nutritional states of the WT, it is apparent that most of the proteome is nutrient-independent with only 37 proteins not identified in both states simultaneously (Fig. 3A). For a quantitative assessment of the measured protein abundances, data were further filtered using a *t* test to analyze only hits that showed statistically significant differences in abundance for data in their respective comparison. A total of 242 of the 1,492 (16.2%) overlapping proteins found in both nutritional states of the WT passed this filtering, indicating that the expression levels of at least 16% of members of the proteome depend on nutrient availability. These data resemble previous label-based iTRAQ quantification analyses, which also indicated nutrient-dependent proteome changes of up to 12% (31). However, comparison of the Δ*saGPN* proteomes with their corresponding WT strains indicated that the amount of significantly altered proteins was approximately two times as high for the knockout strains, with 546/1,413 (38.6%) for nutrient-rich and 445/1,467 (30.3%) nutrient-starved conditions, respectively (Fig. 3A). While this analysis reflected statistically significant changes, we also verified that the changes in protein levels were appropriately high by additionally filtering for proteins that showed a change of at least 50% in their respective comparison. This further filtering for largely altered expression levels (>50%) yielded 83 proteins for the WT_starved_/WT_rich_, 134 for the WT_rich_/Δ*saGPN*_rich_ and 127 for the WT_starved_/Δ*saGPN*_starved_ comparison (Fig. 3B). Heatmap analysis of this filtering shows that the distribution of over- and underregulated proteins is similar between WT states, whereas the knockout seems to have a more pronounced effect on downregulation. Nevertheless, the absence of the GPN-loop GTPase has profound effects on protein expression, even when only highly significant and major changes are considered.

Evaluation of the gene ontology (GO) terms of the proteins shown in the heatmap analysis (Fig. 3B) revealed that a large variety of different protein functions are affected by the *saGPN* knockout (e.g., ArlB, Uniprot: Q4J9K5), pointing to a global role of *Sa*GPN in protein homeostasis (Table S4). However, given the unaffected growth rate of the *saGPN* deletion strain, these changes in protein levels may correspond more to different lifestyles of this archaeal organism, e.g., due to the observed loss of motility (Fig. 2).

### Biochemical and biophysical characterization of *Sa*GPN

**Fig 4:**
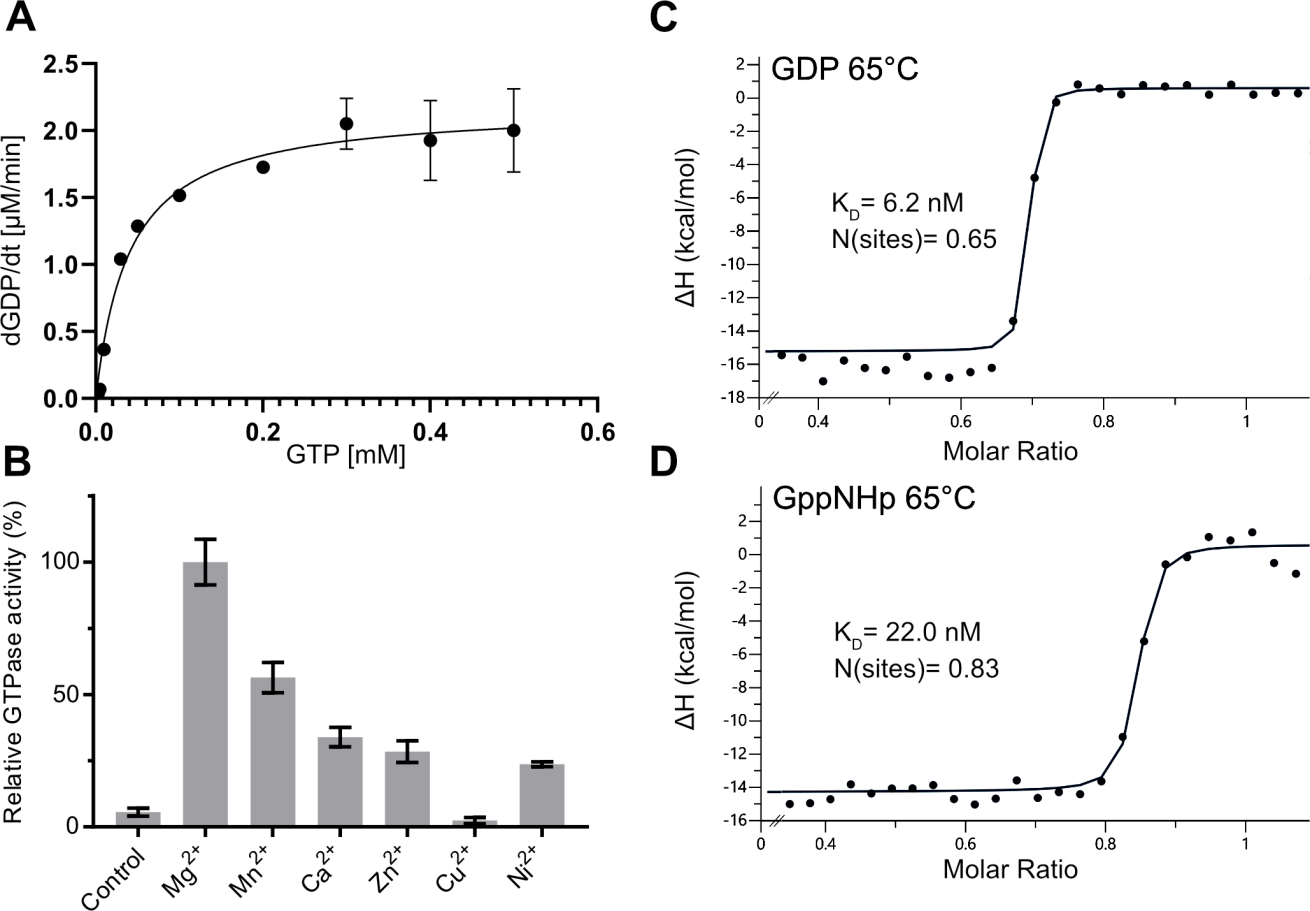
Despite showing low GTPase activity *Sa*GPNs nucleotide binding is very efficient. (**A**) Michaelis-Menten plot of *Sa*GPN activity at 65°C, revealing an activity of 6.4 nmol hydrolyzed GTP per min and per mg of protein. Data represent mean ± s.d. of *n* = 3 replicants. Error bars (standard deviation) of some data points are too small to be visualized. (**B**) Effect of different bivalent metal ions on GTPase activity, which is highest with Mg^2+^. Data represent mean ± s.d. of *n* = 3 replicants. (**C**) ITC measurement of *Sa*GPN at 65°C (to avoid premature protein degradation) with titration against GDP, showing a substrate affinity of *K*_*D*_ = 6.15 nM for GDP. (**D**) Same measurement with titration against GppNHp, showing a substrate affinity of *K*_*D*_ = 22.0 nM for GppNHp. Values are calculated from triplicate measurements.

In size-exclusion chromatography, purified *Sa*GPN (30.5 kDa) eluted as a monodisperse peak at a volume corresponding to a molecular weight of ∼60 kDa, suggesting *Sa*GPN forms a dimer in solution (Fig. S1A and B). GTPase activity of *Sa*GPN was highest in the presence of Mg^2+^ at the optimal growth temperature (75°C) of *S. acidocaldarius* (Fig. S1C). However, this was not applicable for all experiments, as mechanical or chemical stress could result in an onset of protein aggregation at 75°C, which is why the temperature was adjusted to 65°C for kinetic experiments if not stated otherwise. *Sa*GPN preferred Mg^2+^ as the cofactor over other tested metal ions (Fig. 4B). To determine the GTP hydrolysis kinetics, *Sa*GPN was incubated with 0–500 μM GTP at 65°C in the presence of Mg^2+^. The calculated GTP hydrolysis was plotted against the GTP concentration and data were fitted with the Michaelis-Menten equation. The GTPase activity of *Sa*GPN had a *K*_*M*_ of 40.48 µM and a *V*_max_ of 6.4 nmol·mg^−1^·min^−1^ (36.4 nmol·L^−1^·s^−1^), respectively (Fig. 4A).

Nucleotide binding efficiency of *Sa*GPN was investigated by employing isothermal titration calorimetry (ITC), yielding dissociation constants (*K*_*D*_) in the low nanomolar range for all nucleotides investigated. To avoid protein degradation throughout the measurements, nucleotide affinity was determined at 65°C, revealing *K*_*D*_ values of 6.15 and 22.0 nM for GDP and the non-hydrolyzable GTP derivative GppNHp, respectively (Fig. 4C and D). At 25°C, the nucleotide affinity is even higher without a significant disparity for triphosphate nucleotides, indicating that GppNHp is a suitable substitute for GTP (Fig. S1D, E and F). Notably, dimerization of *Sa*GPN is very stable against high dilutions of ≤15 nmol as shown by massphotometry, as no dissociation was observed during measurements (Fig. S1G).

### *Sa*GPN overall structure

The structure of *Sa*GPN was determined from a monoclinic crystal form at a resolution of 1.8 Å in its GppNHp-bound state (PDB: 7ZHF) and solved by molecular replacement, covering residues Y2-A240, with one molecule in the asymmetric symmetry unit (a.s.u.) forming a physiological dimer when crystal symmetry is applied. In addition to the GppNHp structure, we have succeeded in solving the *Sa*GPN structure in its GDP-bound state from an orthorhombic crystal form (PDB: 7ZHK, Y2-A240) at a resolution of 2.4 Å. *Sa*GPN for both crystal forms was purified before in a nucleotide-free state and co-crystallized with the respective nucleotide (Fig. S9C). The GDP-bound structure contains three molecules per a.s.u., where monomers A and C form together a physiological dimer. Likewise, monomer B forms a dimer with its crystal mate B′. The difference between both *Sa*GPN dimers in this crystal form is minor, as a superposition of the dimers yields an r.m.s.d value of only 0.24 Å (445 Cα atoms). Interestingly, the *Sa*GPN dimer interface shows local heterogeneity near the GTP binding site (Fig. S12B through D). In monomers A and B, a double hydrogen bond between N70 of the GPN motif with Q106′ is formed, whereas altered χ_2_ angles of N70′ and Q106 of monomers C and A, respectively, are consistent only with the formation of a single H-bond.

**Fig 5:**
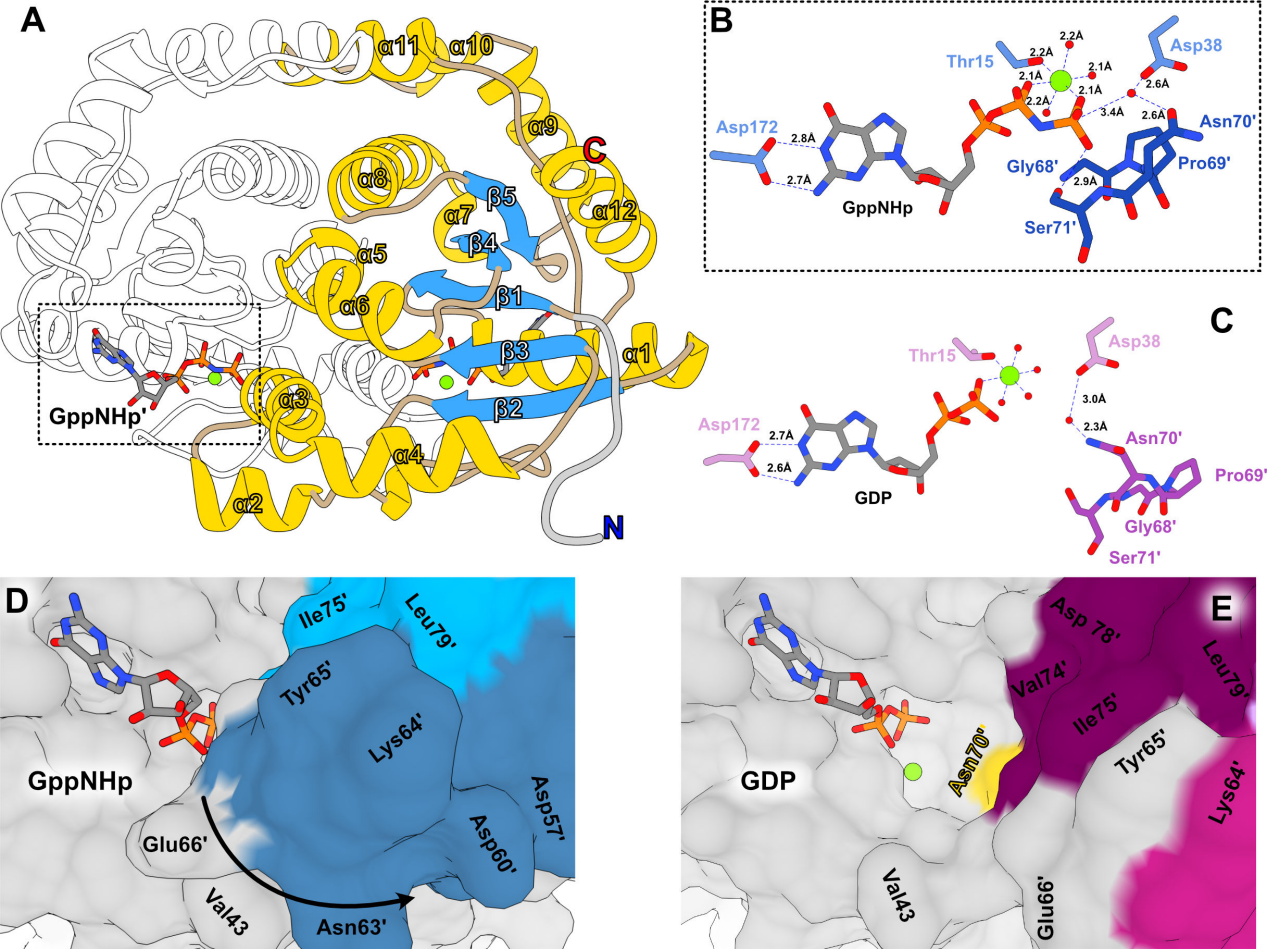
Overall structure of *Sa*GPN and its substrate pocket. (**A**) Structure of *Sa*GPN(GppNHp) with numbering of secondary structure elements on one protomer. Helices and helical turns are colored in yellow (although helical turns of less than four amino acids are not numbered), β-sheets in blue, non-secondary regions in wheat, and the His-tag in gray. (**B**) Coordination of GppNHp in the substrate pocket with most important side chain interactions, including the near perfect Mg^2+^ octahedron and the catalytic active water coordination, with Mg^2+^ in green and waters in red. Primed residues refer to the second protomer. (**C**) Analogous coordination of GDP in the substrate pocket, revealing the difference in Mg^2+^ coordination as well as the increased distance toward the catalytic active water. (**D and E**) Surface view of the substrate pocket in its GppNHp (closed, PDB: 7ZHF) and its GDP state (open, PDB: 7ZHK) with coloring analogous to Fig. 6A for better orientation.

The general structure of SaGPN involves 12 α-helices and a five-stranded, parallel β-sheet that is sandwiched by α6/α8 and α1/α12, respectively (Fig. 5A). Moreover, several small helices and helical turns consisting only of a few amino acids can be found throughout the whole structure, including α5, α7, and α10. The crystal structures of both crystal forms show homodimers of *Sa*GPN, concurring with the SEC elution volume (Fig. S1). The dimer interface emerges alongside the surface of helices α3, α5, α8, and α11, whereby α8 contributes substantially to the dimeric assembly due to its central position allowing the formation of a hydrophobic pocket. This pocket is covered by a roof-like scaffold formed by α9–α11/α11′–α9′ (residues E178-N212) laying orthogonally over α8 helices (residues P143-R159) and parallel to the α2 (residues V58-Y65), the so-called skid region, located at the opposite end. The eponymous GPN motif (residues G68-N70) is found at the base of α3 (residues N70-L81), which is involved in activation of the inline attacking water molecule (N70) and nucleotide coordination at the substrate pocket of the second protomer. Notably, the water that is suitably positioned for inline attack has still a distance of 3.4 Å from the γ-phosphate of the GppNHp substrate mimic. Accordingly, additional conformational changes for the GPN motif may be feasible during hydrolysis of GTP itself. Nucleotide binding is mainly guided by interactions with the backbone; however, side chain interaction of D172 and K170 seems mandatory for coordination of the base and the ribose, respectively. Additionally, the Walker A motif (GxxxxGK[T/S], x = any amino acid) takes part in nucleotide binding, whereas K14 is responsible for the coordination and orientation of the β/γ phosphates of the nucleotide; T15 is the only proteinaceous part of the octahedral Mg^2+^ coordination sphere (Fig. 5B) (32). Mg^2+^ coordination is also supported by the Walker B motif (hhhhD/E, h = hydrophobic), which interacts indirectly with the Mg^2+^ ion over a water bridge to D102; the latter residue also forms a hydrogen bond with the hydroxyl moiety of T15.

As mentioned above, we have succeeded in solving the *Sa*GPN structure in its GDP-bound state, allowing comparison between both states as well as with the only other known archaeal GPN-loop GTPase structure, *Pa*GPN from *P. abyssi* (see Discussion). Apart from the missing γ-phosphate the binding mode of Mg^2+^·GDP to *Sa*GPN is comparable to that of Mg^2+^·GppNHp (Fig. 5C). Interestingly, we observe a water molecule coordinated between D38 and N70′ (primed residues mark the second protomer) close to the position observed before for the inline attacking water molecule of the GppNHp-bound state thus corroborating the role of N70′ of the GPN motif in positioning the water molecule for catalysis. However, due to the loss of the γ-phosphate interaction, the GPN loop detaches from the nucleotide, resulting in an opened substrate pocket around the phosphates (Fig. 5D and E). Interestingly, attempts to derive a GDP·AlF_4_^−^- or GDP·P_i_-bound form that mimics the transition state or product of hydrolysis from the GDP-bound form by soaking or co-crystallization failed, as we only got structures of the GDP-bound state. Accordingly, the GDP-bound state of *Sa*GPN apparently represents a conformation, whose stability exceeds those of the non-covalently linked GDP·AlF_4_^−^- or GDP·P_i_-bound states.

### Allosteric triggering of *Sa*GPN by bound nucleotides

**Fig 6:**
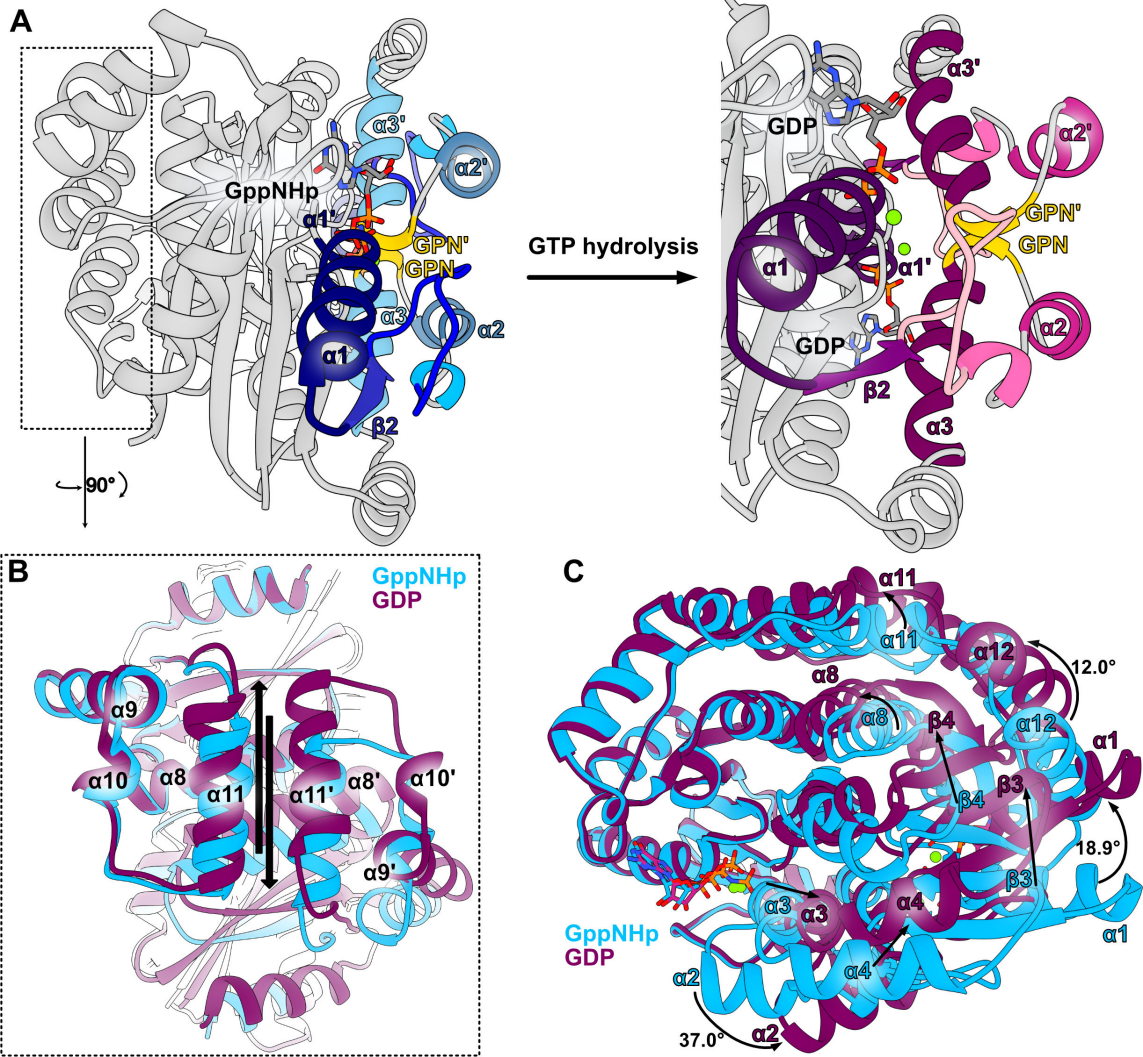
Allosteric changes of *Sa*GPN upon nucleotide hydrolysis observed in crystal structure. (**A**) *Sa*GPN skid region with corresponding allosteric changes upon nucleotide hydrolysis, revealing the push-out movement of the α2 skids. Secondary structure elements are colored in a blue gradient (GppNHp state) or purple gradient (GDP state) for better orientation, and GPN motif in yellow. (**B**) Top-down view on the roof helices with arrows indicating displacement between both states upon nucleotide hydrolysis. (**C**) Overall structure alignment of both nucleotide states, revealing major allosteric changes between states on outside helices as well as inside β-sheets. Most prominent helix shifts as calculated by Chimera are given in degrees.

While the quaternary structures of the GppNHp and GDP dimer states are clearly different for *Sa*GPN (Fig. 6, r.m.s.d values of 2.3 Å over 442 Cα atoms after superposition), structural changes of the different binding states for *Pa*GPN are only subtle, as shown by an r.m.s.d value of 1.0 Å (453 Cα atoms) between GTP- and GDP-bound states (1YR8 vs 1YRB). Considering the monomer itself, comparisons between *Sa*GPN and *Pa*GPN reveal relatively small differences for the GDP-bound state, 1.3 Å for 179 Cα atoms, which are increased to 1.6 Å (181 Cα atoms) for the GTP/GppNHp-bound states. Accordingly, the GDP states of *Sa*GPN and *Pa*GPN are closely related, whereas the GTP-bound form of *Sa*GPN differs from its GDP state as well as the *Pa*GPN states described before. Accordingly, these large allosteric changes undergone by *Sa*GPN upon nucleotide hydrolysis are mostly rigid body-like motions, which are driven by a scissors-like movement of the α2 (residues V58-Y65) skid region (Fig. 6A). Achieving this scissors-like motion seems to involve many local changes throughout the whole GTPase assembly: the roof helices shift against each other (Fig. 6B) with the C-terminal α10 (residues S190-E194) tails being pushed upwards, while α8 helices (residues P143-R159) move toward and α9/ α10 helices (residues E178-D188/S190-E194) drift away from each other. Moreover, α1 (residues T15-N27) is stretched away from the skids and α3 (residues N70-L81) bases move closer to each other, resulting in a convergence of the GPN motifs. Overall, the allosteric movement of both *Sa*GPN states takes primarily place alongside the dimer interface. This also includes α1 (residues T15-N27) and α12 (residues L228-L239), which are not part of the dimer interface yet contribute significantly to the scissors motion, with displacements of 18.9° and 12.0°, respectively (Fig. 6C). Additionally, the γ-phosphate-GPN interaction is lost after hydrolysis, resulting in a swivel-off motion of the GPN-loop including the preceding α2 skids (residues V58-Y65), thus relaxing the whole assembly.

### In solution analysis of *Sa*GPN allosteric movements

**Fig 7:**
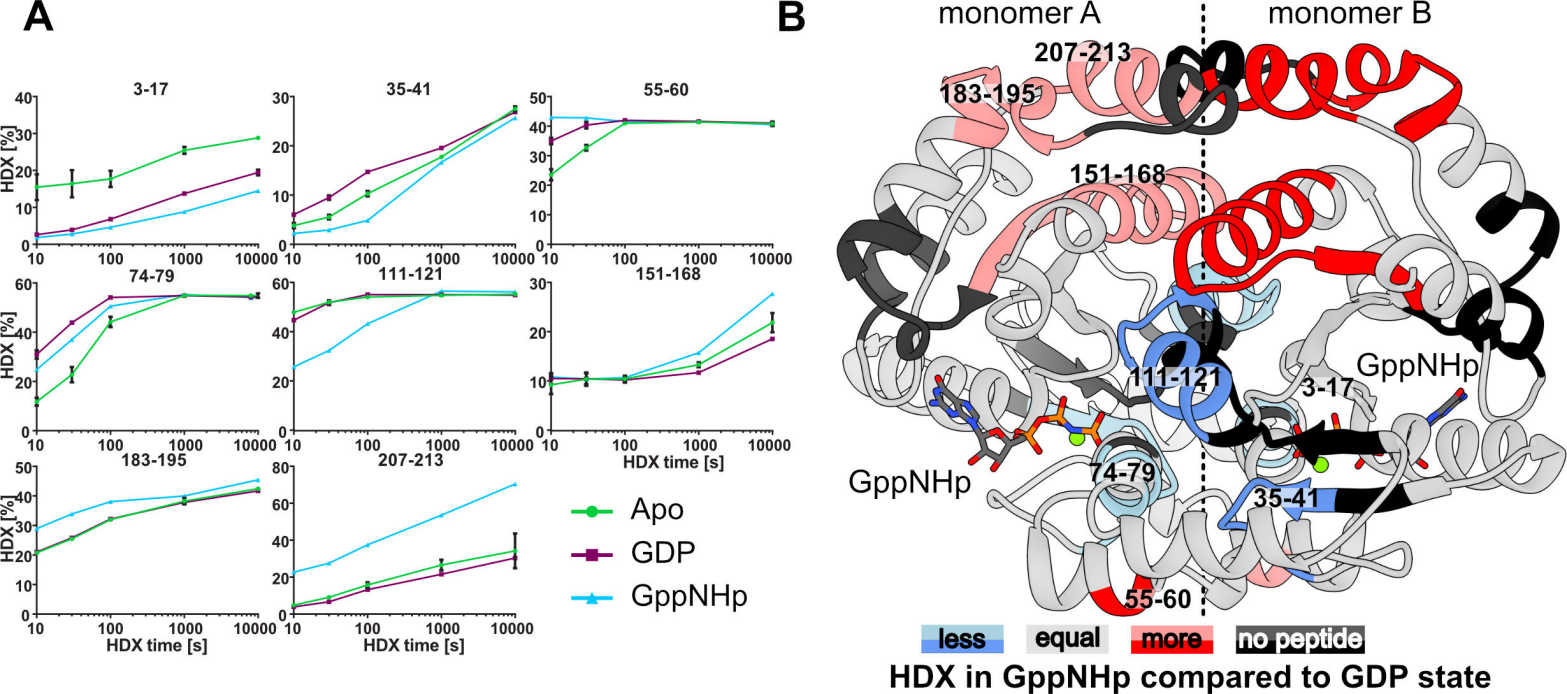
Allosteric changes of SaGPN upon nucleotide hydrolysis observed in solution. (**A**) HDX of representative SaGPN peptides covering regions of difference between the apo state (green circles), GDP-bound state (purple squares) and GppNHp-bound state (blue triangles). Data represent mean ± s.d. of *n* = 3 measurements. (**B**) Difference in HDX between GppNHp and GDP state mapped on SaGPNs GppNHp structure (cumulative meaning that any HDX that happened of the respective region was mapped onto the structure with its highest difference). HDX data show that differences between both states are most prominent in roof region, core elements, and substrate coordinating/hydrolyzing regions, which goes along with most allosteric changes observed in the crystal structure. Numbers refer to amino acid position.

We furthermore probed the nucleotide-dependent changes of *Sa*GPN in solution by hydrogen-deuterium exchange (HDX) coupled to mass spectrometry. Coordination of the GDP and GppNHp nucleotides by *Sa*GPN is evidenced by a reduction in HDX in the G1 motif (peptide F3-L17), compared to the apo state (Fig. 7A and B; Fig. S4 and S5). Further conformational changes induced by GDP encompass helices α2 and α3 (peptides Y55-D60 and V74-L79, Fig. 7B; Fig. S4), both of which exhibit elevated HDX, and helix 6 and the subsequent strand β5 displaying reduced HDX (Fig. S4). Binding of GppNHp to *Sa*GPN evokes even more pronounced perturbations in HDX (Fig. S5). In addition to helices α3 and α4 incorporating more deuterium similar to the GDP-bound state, the roof-constituting helices α9-α11 (residues E178-N212) specifically for GppNHp/*Sa*GPN incorporate more deuterium (peptides L151-L168, R183-L195, and K207-L213; Fig. 7A and B). The changed incorporation rates go along with the significant movement of the roof helices (residues E178-N212) observed in the crystal structure. Most notably, upon GTP hydrolysis, helix α11 (E200-N212) moves with its center-of-mass (COM) by 3.0 Å relative to its core *Sa*GPN domain. This and the changed packing of the core domains in the dimer cause an overall displacement of the α11 helices by 6.3 Å (COM) relative to each other with chain A as point of reference (Fig. 6B). Interestingly, the roof top helices of *Sa*GPN are not involved in crystal contacts, neither in the GDP- nor in the GppNHp-bound form. On the contrary, parts of helices α6 (peptide A111-S121) close to the subunit interface incorporate less deuterium, suggesting an altered topology of *Sa*GPN in presence of GppNHp. The large conformational changes between GDP and GppNHp-bound *Sa*GPN are also reflected in a direct comparison of their HDX behaviors (Fig. S6). Notably, while changes in HDX can be interpreted as changes in the structural assembly or movement of the region affected, the converse is no confirmation for a lack of conformational changes as exemplified by the skid region.

### GTPase activity and phosphorylation of *Sa*GPN are not required for motility

In order to investigate the impact of nucleotide binding by *Sa*GPN *in vivo*, we conceived of mutants that, based on our crystal structure should be defective in GTP binding or hydrolysis (i.e., K14, D38A, D102A, and GPN-AAA). All of these mutants were expressed in *Escherichia coli*, purified, and tested for their GTPase activity, which was almost completely lost in all mutant proteins (Fig. S2A). When complementing the Δ*saGPN* strain with these *Sa*GPN mutants, all mutant proteins could be expressed in *S. acidocaldarius* (Fig. S2C). Surprisingly, compared with the control, the swimming defect could partly be restored by these *Sa*GPN mutants, although they were catalytically inactive (Fig. S7A). MANT-GTP binding assays indicated that these mutants still retained 20–50% of the wild-type GTP-binding capacity (Fig. S7C), suggesting that nucleotide binding is unusually strong for a GTPase, since nucleotides generally fail to bind to G1/Walker A mutants (33). Apparently, *Sa*GPN functions *in vivo* independently from its GTPase activity.

Notably, a phosphoproteomics study of *S. acidocaldarius* demonstrated that *Sa*GPN can be apparently phosphorylated at Y59 (26). A regulatory function for Y59 by (de)phosphorylation can be ruled out as shown by the Y59F mutants swarming assay (Fig. S7B). The *in vivo* and *in vitro* function of *Sa*GPN is unaffected by the removal of this putative site of phosphorylation (Fig. S7B).

## DISCUSSION

GPNs are present in most organisms, in archaea occurring mostly as a single paralog and in eukaryotes in triple paralogs (GPN1–GPN3), where they perform non-redundant essential functions (14). However, little is known about this class of GTPases compared to other guanosine nucleoside phosphate hydrolyzing enzymes like small GTPases or G proteins. In 2007, GPNs were introduced by Gras et al. (13) as a self-activating, homodimeric GTPase family alongside the first GPN crystal structure from *P. abyssi*. Our data now provide new insights into this understudied protein family of GPN loop GTPases.

We were able to solve crystal structures of *Sa*GPN loaded with either an analog of GTP or the hydrolysis product GDP. These structures reveal major changes of the GPN quaternary assembly upon nucleotide hydrolysis, which have not been observed before. After GTP hydrolysis and loss of the γ-phosphate, the substrate pocket switches from a closed into an open state by losing the interaction to the second protomer’s GPN motif. When making a comparison between *Sa*GPN and the only other characterized archaeal GPN, *Pa*GPN (sequence identity 33%) from the hyperthermophile *P. abyssi*, we found that one of the most prominent differences is found in the region of the roof helices. These helices differ in length and, most importantly, adopt alternative orientations when comparing the *Pa*GPN and *Sa*GPN homodimers (Fig. S11). Additionally, the so-called skid region comprising the α2-helix undergoes a prominent conformational change upon transition from the GTP- to the GDP-bound state of *Sa*GPN, but not of *Pa*GPN (Movie S1; Fig. S11). This lack of observable conformational changes for *Pa*GPN may be caused by the enhanced hyperthermophilic nature of *P. abyssi* (growth optimum at 96°C compared to 80°C for *S. acidocaldarius*), which necessitates thermal activation. For example, conformational differences between heat-activated and non-activated enzymes have been found for the dimeric homoserine dehydrogenase from *Sulfurisphaera tokodaii* (34). Interestingly, the inactive homoserine dehydrogenase bound a non-cognate NADP^+^ cofactor and underwent conformational changes near its active site with release of this cofactor only upon heat activation. Although in the *Pa*GPN structures, a partial closing of the catalytic cavity like in *Sa*GPN was not observable, minor changes in the quaternary structure could still reflect the onset of allosteric changes. Nevertheless, other factors like crystal packing or harboring a second Mg^2+^ ion in the *Pa*GPN GDP structure (PDB: 1YRB) may alternatively cause arrest of *Pa*GPN in a single quaternary state.

Additionally, GPNs, including *Sa*GPN, are annotated as GTPases activated by dimerization (GAD), which are G proteins known for exhibiting GTPase activity in the absence of GEFs or guanosine activation protein (GAP) (3). However, while dissociation constants of GADs to guanosine nucleotides are reported to be within the low µM range, *Sa*GPN features an exceedingly high affinity for guanosine nucleotides in the low nM range, matching the values of GTPases employing GEFs (3, 10, 35, 36). Furthermore, GPNs are dimeric regardless of their nucleotide-bound state, distinguishing them from GADs (10, 13). Accordingly, GPNs are different from most other G proteins and form a distinct class of their own as defined by the major part of the IPR004130 family. For example, Q106 of *Sa*GPN is the structural pendant of Q61 from the switch II region of the small G-protein Ras that contributes to catalysis by interacting with the γ-phosphate via a bridging water molecule. In the *Sa*GPN dimer, Q106 is placed in its interface region. Here, the side chain of Q106 H-bonds to that of N70′ in the GDP-bound state, whereas it flips to form a staggering interaction in the GTP-bound state. Despite conservation, Q61 in Ras exerts a different function than Q106, as in the *Sa*GPN dimer, this residue entangles the two nucleotide binding states via the catalytically relevant N70 residues. Notably, the comparably low GTPase activity appears to be a hallmark of archaeal GPN-loop GTPases as shown by turnover rates of 6.4 nmol/min/mg (65°C) for *Sa*GPN and 12 nmol/min/mg (80°C) for the orthologous *Pa*GPN (3, 13). These turnover rates may still allow a biological function for archaeal GPN-loop GTPases in the absence of canonic GAPs and/or GEFs, although the complete absence of at least an allosteric partner enhancing archaeal GPNs GTPase function cannot be ruled out. The latter implies that the term “self-activated” as originally introduced by Gras et al. might not be appropriate. Another issue is given by the fact that kinetic experiments for *Sa*GPN had to be performed at 65°C for technical reasons, i.e., below the optimal growth temperature of 75–80°C of *S. acidocaldarius*. Accordingly, nucleotide exchange rates, affinities, and the Michaelis-Menten-like kinetic analysis of GTP hydrolysis might be affected. However, due to the apparently low intrinsic GTPase activity and high guanosine nucleotide affinities, both nucleotide-bound states of *Sa*GPN have considerable half-lives. This may be particularly relevant for the GTP-bound state to exert a potential function in regulation and protein-protein interaction. For comparison, eukaryotic GPNs are not only involved in sister chromatid cohesion (human) and, like a chaperone, the assembly of the 12-subunit RNA polymerase II (*S. cerevisiae*), but also in other biological processes such as mitochondrial homeostasis and ribosome biogenesis (14, 16, 37, 38). Given this broad range of functions for eukaryotic GPNs, archaeal GPNs like *Sa*GPN with their conformational switching between GTP- and GDP-bound states may exert a similar type of function, e.g., by acting as a chaperone during multi-protein assembly.

Deletion of GPNs is known to be lethal in eukaryotes (13, 14, 18), but interestingly not in the case of the crenarchaeote *S. acidocaldarius*. This implies that the biological context of archaeal GPNs differs from that of their eukaryotic counterparts. The deletion of *saGPN* in *S. acidocaldarius* yields a phenotype with a highly reduced motility despite lacking any apparent growth defect. The motility phenotype is not strictly dependent on the catalytic capability of *Sa*GPN to hydrolyze GTP, as different mutations in the G-box, which cause impaired nucleotide binding and/or lack the ability to hydrolyze GTP, resulted only in gradually diminished motility (Fig. 2; Fig. S7). These observations suggest that the intrinsic GTPase activity of *Sa*GPN is dispensable for motility of *S. acidocaldarius*. However, it is unclear whether GTPase activity is of relevance to other processes that were not investigated in this study. Moreover, we showed that a mutation within the α2-helix, Y59D, affects swarming similar to the G-box mutants besides decreasing catalytic activity by ~70%. Nevertheless, this correlation between GTPase activity and motility may be serendipitous as G-box mutants lack GTPase activity while still exhibiting motility. Thus, the decreased motility of the surface-exposed Y59D mutant may be due to distorted interactions with unknown downstream partners, which control synthesis, assembly or, activity of the *S. acidocaldarius* archaellum.

A broader biological function of *Sa*GPN than its requirement for motility is indicated by our proteomics analysis of the *saGPN* knockout in *S. acidocaldarius*. Relative protein quantification by timsTOF of the Δ*saGPN* mutant compared to the wild-type strain revealed an unexpectedly large number of proteins being affected in their levels. Notably, these proteins are assigned to a highly diverse set of biological functions, which are mostly not directly linked to motility. Compared to the pronounced metabolic adaptation of the *S. acidocaldarius* proteome upon starvation (Fig. 3A), the even larger impact of the *saGPN* deletion suggests a key role of *Sa*GPN in protein homeostasis of *S. acidocaldarius*. Interestingly, the *saGPN* deletion also resulted in a complete loss of an ortholog of the universal stress protein family, UspD (Uniprot: Q4JA32). UspD was undetectable in the proteome of the *saGPN* deletion strain, although it was highly abundant in all WT samples (Table S4). Interestingly, UspD has only very limited sequence identity (8–11%) to the known Usp proteins of *S. acidocaldarius*, UspA-UspC, although it shares the typical Usp fold with the latter (Fig. S8). Given that *Sa*GPN was found together with UspA in a previous co-IP assay (25), it is notable that UspA levels are almost unaffected in the *saGPN* deletion strain.

**Fig 8:**
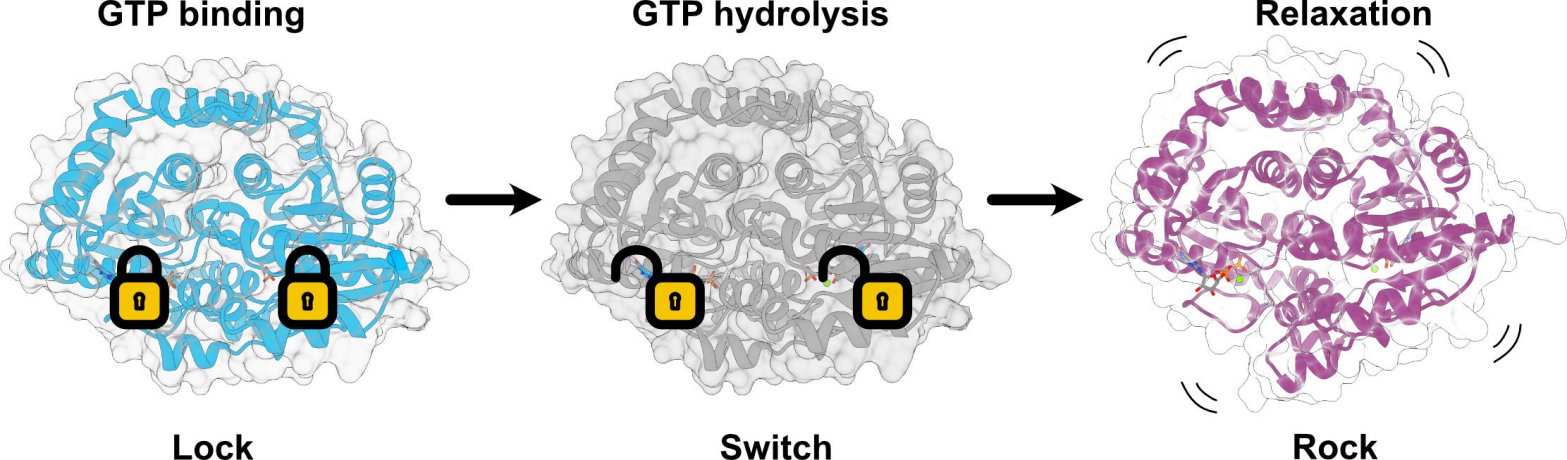
Lock-switch-rock (LSR) mechanism of *Sa*GPN. After GTP binding and before nucleotide hydrolysis, *Sa*GPN enters a locked state and stores tension throughout the structure. GTP hydrolysis leads to a loss of interaction in the GPN loop, which allows the transition from the locked state to a relaxed state in which a rocking motion is performed, stretching the GTPase assembly. Since these three steps represent the main catalytic states, we developed the lock-switch-rock mechanism, or LSR mechanism, for easy visualization and reference.

Since we were able to obtain snapshots of *Sa*GPNs catalytic cycle, we propose a mechanism, which we call “lock-switch-rock” mechanism (Fig. 8) that assigns a catalytic function to the conserved Gly-Pro-Asn motif of GPN-loop GTPases. The GTP-bound state as visualized by our structure of the *Sa*GPN·GppNHp complex (7ZHF) shows a partial closure of the nucleotide-binding site by the embracing interaction between the GPN motif from the opposing *Sa*GPN protomer and the γ-phosphate (Fig. 5). As a consequence, a water molecule that is coordinated between D38 from the G2 motif and N70′ of the GPN motif is now found in an inline attack position for initiating the S_N_2 reaction at the γ-phosphate. When deprotonated, D38 acts here as a general base for transient formation of the attacking hydroxide nucleophile like the corresponding residue in the G2 box of other SIMIBI GTPases (13). Overall, the GTP-bound state of GPN-loop GTPases with its closed GPN-lid corresponds to a ‘locked’ state. The quaternary structure changes in a ‘rock’-like motion upon the ‘switch’ event, i.e., nucleotide hydrolysis and phosphate release. Its trigger may be other protein interaction partners as exemplified by eukaryotic GPN-loop GTPases during RNA polymerase II assembly, but these factors, if they exist, are yet not known for *Sa*GPN. Unfortunately, efforts to crystallize a transition mimic of the *Sa*GPN·GTP complex, i.e., the “switch” state, failed. Accordingly, we do not yet know the exact nature of the “switch” state of GPN-loop GTPases as switching may be triggered by either the hydrolysis event or the release of phosphate from the *Sa*GPN·GDP·P_i_ state. In any case, the “rocking” motion causes an increased distance between the nucleotide and the GPN loop besides changes at the distal side of the GPN dimer along the roof helices. Interestingly, the interaction between D38/N70′ with a water molecule is even maintained in the GDP-bound form. This corroborates the notion that N70 of the GPN loop is essential to position the attacking water in the GTP-bound state, while the Gly-Pro motif caps helix α3′ at its N-terminus, so that it points toward the γ-phosphate and D38 for stabilizing dipole-ion interactions.

Overall, hydrolysis to GDP with phosphate release removes these intimate interactions between the GPN motif/helix α3′ and the γ-phosphate and subsequently drives the switching from a locked state toward a relaxed state with a concurrent rocking motion that affects the whole GPN dimer (Fig. 8; Movie S1).

## MATERIALS AND METHODS

### Strains and growth conditions

*S. acidocaldarius* wild-type strain MW001 and all derived mutants in this study (Table S1) were cultivated at 75°C in Brock basal medium (pH 3.0–3.5, all salts for the media are from Roth) supplemented with 0.1% (wt/vol) NZ-amine (Sigma-Aldrich), 0.2% (wt/vol) dextrin (Roth), and 10 µg/mL uracil (Sigma-Aldrich) (39, 40). For *S. acidocaldarius* strains containing complementation plasmids, uracil was not needed.

### Construction of *S. acidocaldarius* Δ*saGPN* mutant

The plasmid pSVA5117 for the markerless in-frame deletion mutant Δ*saGPN* (Table S1) was constructed as described previously (40). After transformation into *S. acidocaldarius* MW001, positive transformants were screened on first selection gelrite (Roth) plates lacking uracil, then grown on second selection gelrite plates with uracil and 5-FOA (100 µg/mL). Finally, Δ*saGPN* mutants were screened by colony PCR with checking primers (Table S2), and further sequencing.

### Nutrient starvation assays and Western blots

Nutrient starvation assays and Western blots were performed as described (41). Briefly, at an OD_600_ of 0.4–0.5, overnight *S. acidocaldarius* cultures were collected at 75°C and re-suspended in Brock medium in the absence of NZ-amine and dextrin, followed by cultivation at 75°C. Samples for the Western blot analysis were taken at the indicated times.

### RNA isolation and qRT-PCR

Total RNA samples were prepared with *S. acidocaldarius* cultures from nutrient starvation assays and qRT-PCR analysis was performed as described before (25). *secY* is a housekeeping gene, which was used as the reference gene for data normalization.

### Transmission electron microscopy

Δ*saGPN* and MW001 strains were grown in 50 mL Brock’s medium supplemented with 0.1% (wt/vol) NZ-amine, 0.2% (wt/vol) dextrin, and 10 µg/mL uracil. At an OD_600_ = 0.2–0.3, nutrient starvation was done for 4 h to induce archaellation. Afterward, cells were applied on freshly glow-discharged carbon/formvar coated copper grids (300 mesh, Plano GmbH) and incubated for 30 s. This was repeated three times and excess liquid was blotted away. Cells were negatively stained with 2% (wt/vol) uranyl acetate (Ted Pella). Imaging was done with Hitachi HT7800 operated at 100 kV, equipped with an EMSIS XAROSA 20 Megapixel CMOS camera.

### Motility assays

Motility assays were performed as described (40). *S. acidocaldarius* strain Δ*arnA* and Δ*arnR*/*R1* were used as a hypermotile and non-motile control, respectively. All strains originate from previous work (27, 42).

### MANT-GTP binding assays

Binding of fluorescent MANT-GTP [(2′-(or-3′)-O-(N-methylanthraniloyl) guanosine 5′-triphosphate] (Jena*-*Bioscience) to *Sa*GPN was measured by monitoring fluorescence increase upon binding to the protein utilizing a Fluoromax-4 spectrofluorometer (Horiba). The excitation wavelength was set to 285 nm, and the emission wavelength was set to 450 nm. Slit widths for both excitation and emission were set to 10 nm. Binding was measured by incubation of 2.5 µM of *Sa*GPN/variants with MANT-GTP (5 µM) in buffer 50 mM MES pH 6.5, 150 mM NaCl, 5 mM MgCl_2_ (Roth) at 25°C. Fluorescence was corrected for MANT-GTP fluorescence in the absence of proteins. Fluorescence of *Sa*GPN wild-type protein was set as 100% for relative MANT-GTP binding analysis.

### Sample preparation for proteomic mass spectrometry analysis

Strains were grown and subjected to 30 min starvation conditions as described above. Subsequently, 200 µL cell pellet equivalents was lysed in 900 µL urea buffer (8 M urea dissolved in 0.1 M NH_4_HCO_3_). After that, the samples were disrupted using glass beads and a FastPrep-24 (MP Biomedicals) homogenizer (6.5 m/s, three cycles of 30 s, 5 min resting on ice between cycles) with subsequent centrifugation (50 min, 14,000 rpm). Supernatant concentration was determined using Bradford assay and afterwards resuspended with the cell debris to yield full cell samples again. Protein concentrations of full cell samples were estimated to be double the amount of the supernatant concentration. Samples were adjusted to 40 µL (8 M urea dissolved in 0.1 M NH_4_HCO_3_) containing 100–200 mg protein based on the concentration estimation and mixed with 1 µL TCEP (0.2 M) with subsequent incubation (1 h, 37°C, 1,000 rpm). About 1 µL iodoacetamide (0.4 M) was added and the solutions were incubated in the dark (30 min, 25°C, 500 rpm), followed by addition of 1 µL *N*-acetyl-cysteine (0.5 M). Samples were incubated (10 min, 25°C, 500 rpm) and diluted with 10.3 µL urea buffer (6 M urea dissolved in 0.1 M NH_4_HCO_3_), followed by the addition of 1.25 µL Lys C (0.2 mg/mL, 1:400 wt/wt) and digestion (4 h, 37°C). Solutions were diluted with 145.3 µL urea buffer (1.6 M urea dissolved in 0.1 M NH_4_HCO_3_), mixed with 2 µL trypsin (1:100 [wt/wt\), and digested (overnight, 37°C). pH was adjusted to <2 with 2.5 µL TFA (0.1% [vol/vol]). Samples were centrifuged (1 min, 14,000 rpm) and transferred to an equilibrated (0.1% [vol/vol] TFA) Chromabond C_18_ spin column (30 s, RT, 2,000 rpm). Peptides were eluted with 2 × 150 µL elution solution (50% [vol/vol] ACN, 0.1% [vol/vol] TFA), dried in a vacuum centrifuge (45°C, 4,000 rpm) and resuspended in 30 µL acetonitrile-TFA solution (10%[vol/vol], 0.1% [vol/vol]). Samples were then measured using a timsTOF mass spectrometer in collaboration with the MarMass facility of Philipps University Marburg.

### Proteomics data evaluation

Data analysis of the timsTOF data was performed with MaxQuant 2.1.3 (43), sequence data for *S. acidocaldarius* DSM 639 (2,222 entries) were downloaded from Uniprot. MaxQuant parameters for label-free quantification (LFQ) analysis were set to a maximal peptide mass of 4,000 Da, up to three modifications per peptide, up to three missed cleavages and the requirement of MS/MS for LFQ comparisons; all other parameters are set to default. Protein abundances of biological replicates of the Δ*saGPN* strain and the wild type strain were filtered by a two-tailed, homoscedastic Student’s *t* test (*P* < 0.05) using a mean abundance diﬀerence of >10% as second criterion. The resulting lists of proteins were used for protein heat maps after filtering with a mean abundance diﬀerence of >50% and visualization by the pheatmap R package. Gene ontology enrichment analysis was carried out using the GOATOOLS library (44), version 1.2.3, in python. In this gene ontology enrichment analysis, terms are represented for molecular function (MF), cellular component (CC), and biological processes (BP) of the identified proteins. To implement the GOATOOLS library, gene ontology terms (GO-Terms) for *S. acidocaldarius* DSM639 were downloaded using the QuickGO annotation online tool (45). A total of 9,487 annotations were available for *S. acidocaldarius* DSM639 proteins covering 1,548 gene products (70%); the remaining 674 are mostly still assigned as “conserved archaeal proteins.”

### Sequence similarity network and *in silico* analysis

Generation of primary SSN data was done with the EFI-Enzyme Similarity Tool (46) web service (Uniprot Version: 2021_03; InterPro Version 87) based on InterPro Family IPR004130 (GPN-loop GTPases) with an UniRef90 restraint due to the size of the IPR (29). SSNs were generated for *E*-values 10^−40^ and 10^−45^ with subsequent cluster analysis (standard options) and colorization. Analysis and visualization of the generated network were performed with Cytoscape 3.8.2 using the yfiles organic layout (47).

### Expression and purification of *Sa*GPN

Recombinant overexpression of N-terminal His_10_ tagged *Sa*GPN was performed in an expression media containing 10 g/L tryptone (Sigma-Aldrich), 10 g/L NaCl, 5 g/L yeast extract (Sigma-Aldrich) and 12.5 g/L lactose (Roth), employing BL21(DE3) Rosetta cells (37°C, 150 rpm, 18 h). Harvesting (20°C, 5,000 rpm, 20 min) was followed by resuspension in lysis buffer (150 mM NaCl, 50 mM Tris, 15 mM EDTA, pH = 8.0) and cell lysis via French Press. The lysate was centrifuged (18,000 rpm, 20°C, 20 min), the supernatant heat-treated (55°C, 15 min) and centrifuged again (18,000 rpm, 20°C, 20 min) before being loaded to a Ni-NTA column (5 mL). After sample application, the column was washed with nine column volumes wash buffer (150 mM NaCl, 50 mM Tris, 25 mM imidazole (Roth), 15 mM EDTA, pH = 8.0) and eluted with five column volumes elution buffer (150 mM NaCl, 50 mM Tris, 500 mM imidazole, pH = 8.0). The elution was concentrated (<2.5 mL) and applied to a HiLoad 16/60 Superdex 200 pg (GE Healthcare) size-exclusion column, which had been equilibrated with running buffer (150 mM NaCl, 50 mM Tris, 10 mM MgCl_2_, pH = 8.0). Fractions containing *Sa*GPN with a 260/280 nM ratio ≤0.55 were collected, concentrated, frozen in liquid nitrogen and stored at −80°C. Protein concentrations were determined by NanoDrop spectrophotometry for the apo-state; Bradford assays were used to confirm concentrations of the nucleotide-bound *Sa*GPN.

### GTPase assays

GTPase activity of *Sa*GPN was measured using the Malachite green assay (48). For a 600-µL reaction mix at 65°C 60 µL 10× *Sa*GPN (final concentration 10 µM) was mixed with 60 µL 10× GTP stocks and adjusted to 600 µL with a buffer containing 50 mM Tris pH 8.0, 150 mM NaCl, and 10 mM MgCl_2_. For each time point, 60 µL of that mixture was taken and quenched with the Malachite green reaction solution. After incubation for 30 min, the Malachite green solution developed its color and was measured. The absorption at 620 nm was measured and a phosphate standard was used for quantification.

### PP2A phosphatase activity assays

Serine/threonine phosphatase activity assay of PP2A was performed using the artificial p-peptide RRA(pT)VA substrate (Promega) in lysis buffer containing 1 mM MnCl_2_ at 70°C (26). The release of free phosphate from artificial p-peptides was measured using the Malachite green assay as described above (48).

### Crystallization of *Sa*GPN

Crystallization screens were performed as sitting drop experiments with 24.5 mg/mL *Sa*GPN (Saci_1281, Uniprot: Q4J9A7) that includes the N-terminal His_10_-tag (MHHHHHHHHHHLEVLFQGPS) and 3 mM nucleotide in running buffer mixed in a 1:1 ration with the respective crystallization condition, using 0.3 µL of each. Crystallization was observed in different conditions of all JCSG Core suits (NeXtal); however, the crystal used for the structure determination of *Sa*GPN(GppNHp) crystallized in the presence of 200 mM NaCl, 100 mM NaOAc (pH = 4.6) and 30% (vol/vol) MPD (JCSG Core III/G11) after 24 h. *Sa*GPN(GDP) was crystallized in the presence of 200 mM MgCl_2_, 100 mM MES (pH 5.5), and 40% (vol/vol) PEG 400 (JCSG Core II/G3) after 24 h. *Sa*GPN crystallization in the apo-state was also attempted but resulted only in weakly diffracting crystals (resolution > 7 Å) and was therefore not suitable for structure determination. Crystals of the apo state grew in many different conditions of the NeXtal JCSG Core suits I–IV.

### Structure determination of *Sa*GPN

Data collection was performed with the Swiss Light Source at Paul Scherrer Institute in Switzerland. Phasing was done employing molecular replacement using BALBES (GppNHp state) and PHASER (GDP state) of the ccp4 pipeline, followed by model building with PDB-REDO and ARP/wARP (49–53), revealing one molecule per asymmetric symmetry unit for the GppNHp-bound and three for the GDP-bound state. The physiological dimeric state for the *Sa*GPN·GppNHp complex can be generated by applying crystal symmetry. For the *Sa*GPN·GDP complex, the dimer is represented by chains A/C and chains B/B′. Structure refinements were done by multiple rounds of manual model building with Coot followed by phenix.refine (54, 55). For both nucleotide-bound states, we were able to build residues Y2-A240 and some residues of the His_10_-tag, resulting in only 14 disordered amino acids at the C-terminus. For the GDP state, structural imposition of the *Sa*GPN chains reveals an r.m.s.d. of 0.23 Å for chains A vs C and chains B vs C that is only slightly increased to 0.24 Å for chains A vs B. Structural analysis was carried out with PyMOL and visualization with Chimera 1.15 (56, 57). F_o_-F_c_ omit maps showing the density around the nucleotides are shown in Fig. S9. Structural morphing between the two nucleotide-bound states was done by Chimera 1.15, rendering of the resulting poses (Movie S1) by Blender 3.6 (blender.org) and Molecular Nodes (bradyajohnston.github.io/MolecularNodes).

### Isothermal titration calorimetry

ITC experiments were carried out with purified *Sa*GPN concentrated to a 100 µM stock solution, corresponding to monomeric protein, and stored at −80°C as 300 µL aliquots. Sample cell had a volume of 200 µL. For each run, *Sa*GPN aliquots and nucleotide stocks (800 µM) were thawed just prior to measurement. The sample cell of the MicroCal PEAQ-ITC, (Malvern) was heated to 65°C or 25°C, before in total 40 µL of nucleotides was added from the injection syringe using constant stirring (52 injections). Evaluation and visualization of ITC data were performed with the Malvern evaluation software. A 1:1 model was used for fitting and calculating *K*_*D*_/*N*-values. It should be mentioned that the high affinity for guanosine nucleotides caused low data resolution. Accordingly, fittings showed only a moderate significance for calculated *N*-values.

### Hydrogen-deuterium exchange mass spectrometry

HDX-MS experiments on *Sa*GPN were carried out similarly as described previously (58). The samples contained 50 µM *Sa*GPN and 5 mM nucleotides (GDP, GppNHp) in a buffer containing 50 mM Tris-Cl pH 8.0, 150 mM NaCl, and 10 mM MgCl_2_. Preparation of the HDX reactions was aided by a two-arm robotic autosampler (LEAP technologies). For deuterated samples, 7.5 µL of *Sa*GPN solution (with or without nucleotides) was supplemented with 67.5 µL of D_2_O‐containing buffer to start the exchange reaction. After 10, 30, 100, 1,000 or 10,000 s at 25°C, samples (55 µL) were taken from the reaction and mixed with 55 µL of quench buffer (400 mM KH_2_PO_4_/H_3_PO_4_, 2 M guanidine-HCl, pH 2.2) kept at 1°C. About 95 µL of the resulting mixture was injected into an ACQUITY UPLC M-Class System with HDX Technology (Waters [59]). Undeuterated samples of *Sa*GPN were prepared similarly by 10‐fold dilution of the protein solution with H_2_O‐containing buffer. Samples were flushed out of the loop (50 µL) with H_2_O + 0.1% (vol/vol) formic acid (flow rate of 100 µL/min) and guided to a column (2 mm × 2 cm) packed with immobilized porcine pepsin and kept at 12°C for proteolytic digestion. The peptic peptides thus generated were collected on a trap column (2 mm × 2 cm) filled with POROS 20 R2 material (Thermo Scientific) kept at 0.5°C. After 3 min, the trap column was placed in line with an ACQUITY UPLC BEH C18 1.7 µM 1.0 × 100 mm column (Waters), and the peptides were eluted at 0.5°C column temperature using a gradient of H_2_O + 0.1% (vol/vol) formic acid (A) and acetonitrile + 0.1% (vol/vol) formic acid (B) at a flow rate of 30 µL/min as follows: 0–7 min/95–65% A, 7–8 min/65–15% A, 8–10 min/15% A, 10–11 min/5% A, and 11–16 min/95% A. The peptides were ionized with an electrospray ionization source (250°C capillary temperature, 3.0 kV spray voltage) and mass spectra acquired in positive ion mode over a range of 50 to 2,000 *m*/*z* on a G2-Si HDMS mass spectrometer with ion mobility separation (Waters), using Enhanced High Definition MS (HDMS^E^) or High Definition MS (HDMS) mode for undeuterated and deuterated samples, respectively (60, 61). Lock mass correction was implemented with [Glu1]-Fibrinopeptide B standard (Waters). During each chromatographic run, the pepsin column was washed three times with 80 µL of 4% (vol/vol) acetonitrile and 0.5 M guanidinium chloride, and blank injections were performed between each sample. All measurements were carried out in triplicate.

*Sa*GPN peptides were identified from the undeuterated samples with the software ProteinLynx Global SERVER 3.0.1 (PLGS, Waters), using the amino acid sequence of *Sa*GPN, porcine pepsin and their reverted sequences as database, and their deuterium incorporation determined with the software DynamX 3.0 (Waters) as described previously (58).

## Data Availability

Protein structure data are deposited in the PDB and can be accessed via the codes 7ZHF (*Sa*GPN GppNHp structure) and 7ZHK (*Sa*GPN GDP structure). Remaining data are found in the supplementary material.

## SUPPLEMENTAL MATERIAL

Supplemental material mbio.00859-23-s0001.docx Fig. S1 to S12, Tables S1 to S3, and supplemental legends.

Table S4 mbio.00859-23-s0002.xlsx timsTOF data and GO term analysis of S. acidocaldarius WT and saGPN knockout strains.

Table S5 mbio.00859-23-s0003.xlsx HDX data and its analysis.

Movie S1 mbio.00859-23-s0004.mp4 Morph of the SaGPNs&amp;#x2022;GppNHp state towards its GDP-bound state, visualizing the major allosteric changes upon nucleotide hydrolysis.

ASM does not own the copyrights to Supplemental Material that may be linked to, or accessed through, an article. The authors have granted ASM a non-exclusive, world-wide license to publish the Supplemental Material files. Please contact the corresponding author directly for reuse.
