# Supplementary material for: Archaeal GPN-loop GTPases involve a lock-switch-rock mechanism for GTP hydrolysis
Source: mBio. 2023 Nov 14;14(6):e00859-23. doi: 10.1128/mbio.00859-23 (PMC10746158; doi:10.1128/mbio.00859-23)
Supplement: Supplemental material — Fig. S1 to S12, Tables S1 to S3, and supplemental legends. [file mbio.00859-23-s0001.docx]

**Supplementary Information for**

Archaeal GPN-loop GTPases involve a lock-switch-rock mechanism for GTP hydrolysis

Lukas Korf^†,1^, Xing Ye^†,2^, Marian S. Vogt^1^, Wieland Steinchen^1,3^, Mohamed Watad^1^, Chris van der Does^2^, Maxime Tourte^2^, Shamphavi Sivabalasarma^2^, Sonja-Verena Albers^2^, Lars-Oliver Essen^1^

^1^Philipps University, Department of Chemistry, Hans-Meerwein-Strasse 4, 35032 Marburg, Germany

^2^University of Freiburg, Institute for Biology II, Molecular Biology of Archaea, Schaenzlestrasse 1, 79104 Freiburg, Germany

^3^Center for Synthetic Microbiology (SYNMIKRO), Karl-von-Frisch-Strasse 14, 35043 Marburg, Germany

^†^Contribution of both authors is considered equal

**Email:**  essen@chemie.uni-marburg.de

sonja.albers@biologie.uni-freiburg.de

**This PDF file includes:**

Figures S1 to S12

Tables S1 to S3

Legends for Movies S1

SI References

**Other supplementary materials for this manuscript include the following:**

Movies S1

Table S4 (proteomics data)

Table S5 (HDX data)

**
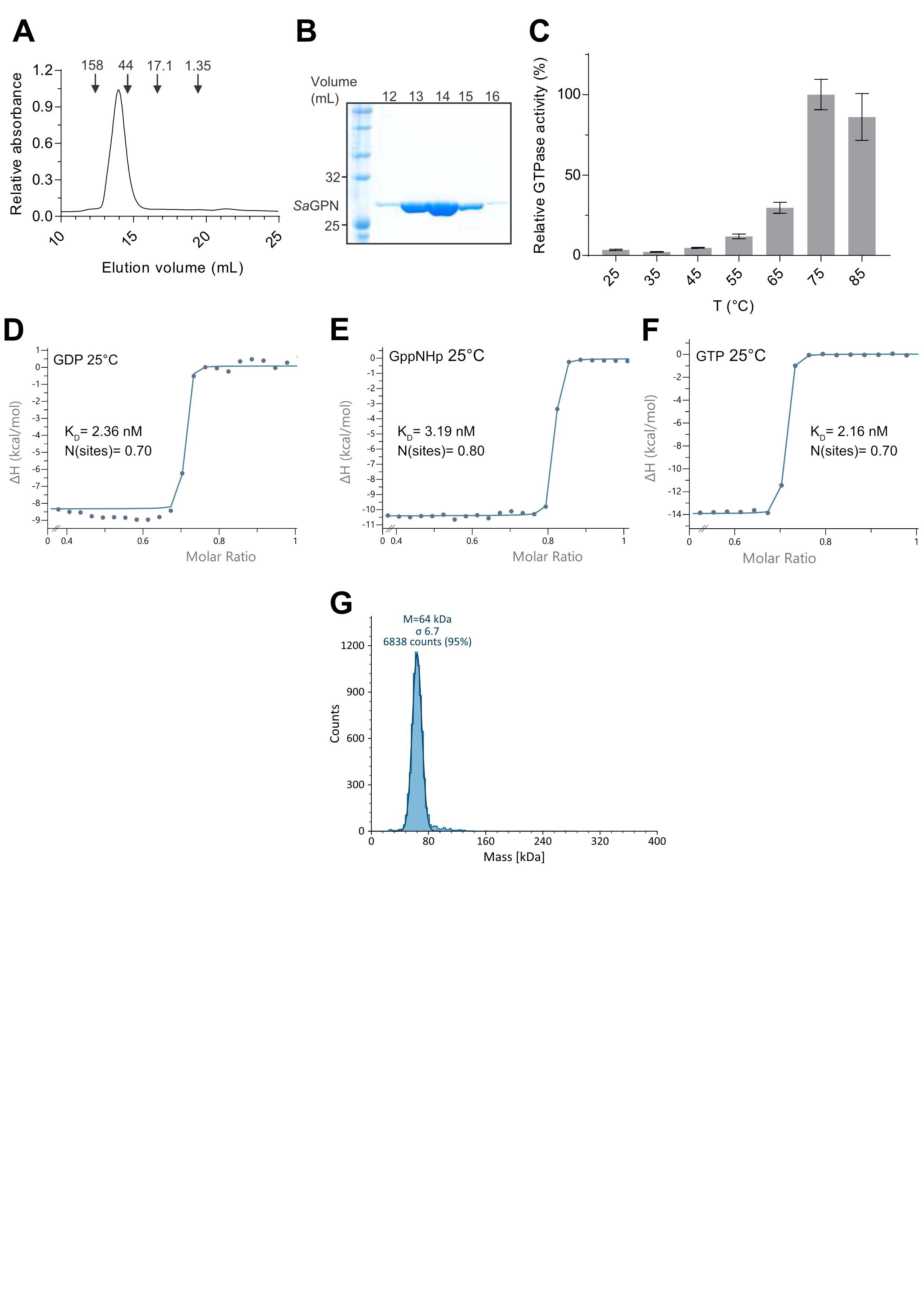
**

**Fig. S1. Biochemical support data to *Sa*GPN. (A)** Purified *Sa*GPN was analyzed on a Superdex 200 10/300 GL column. Molecular weight standard of 158, 44, 17, and 1.35 kDa are indicated. **(B)** Fractions from **(A)** were analyzed by SDS-PAGE. **(C)** Temperature dependency of *Sa*GPN hydrolysis activity. Data represent mean +- s.d. of n=3 replicates **(D)** ITC measurement of *Sa*GPN at 25 °C with titration against GDP, showing a substrate affinity of K_D_ = 2.4 nM for GDP. **(E)** Same measurement with titration against GppNHp, showing a substrate affinity of K_D_ = 3.2 nM for GppNHp. **(F)** ITC measurement of *Sa*GPN at 25 °C with titration against GTP, showing a substrate affinity of K_D_ = 2.2 nM for GTP. This measurement was performed as a single run for check-up only. **(D)** Massphotometer measurement of *Sa*GPN with a final concentration of 50 nmol to check the potential dissociation of *Sa*GPN dimer against high dilution. No dissociation of the dimeric *Sa*GPN could be observed.

**
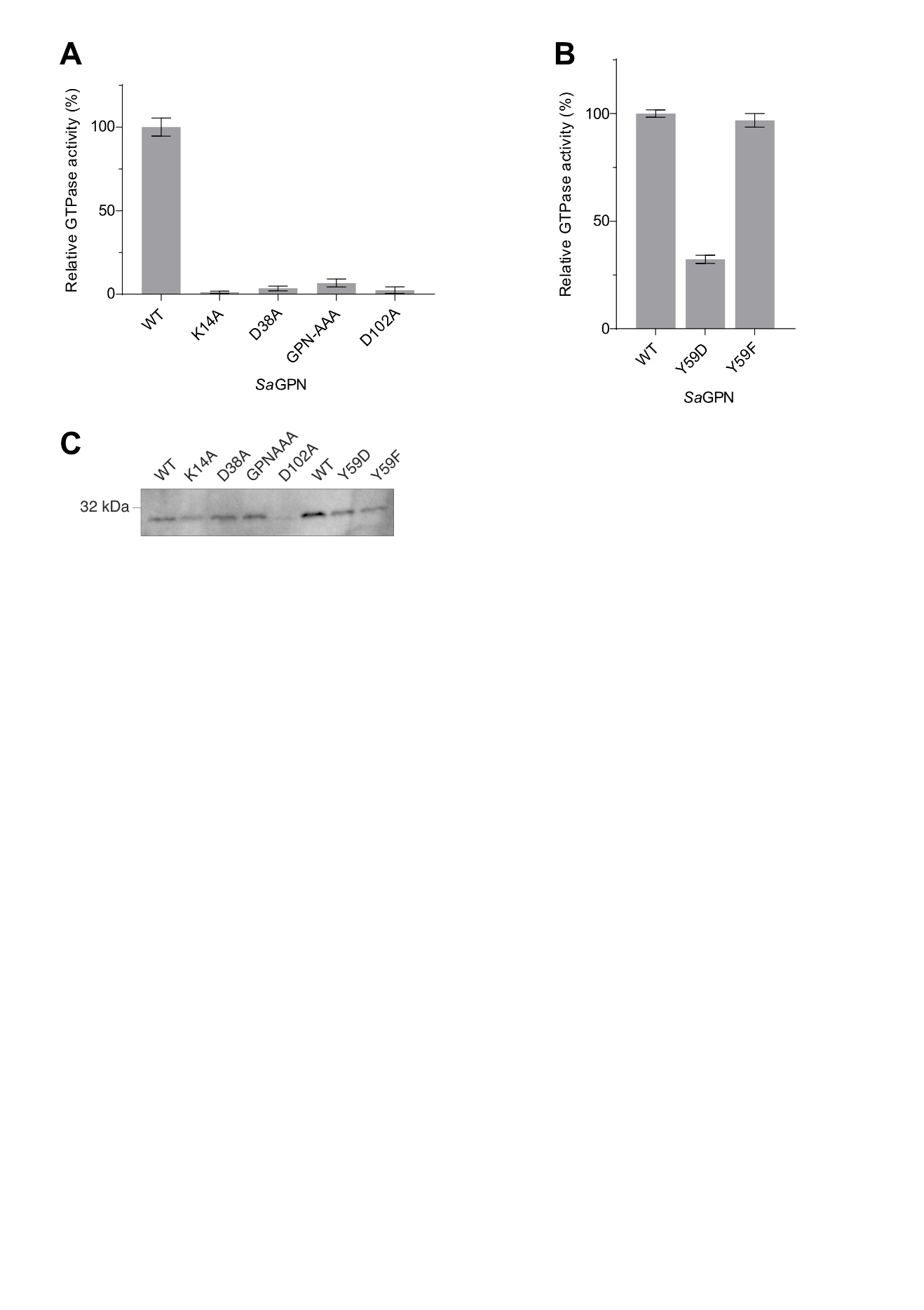
**

**Fig. S2. GTPase activity of *Sa*GPN mutants. (A)** GTPase activity of *Sa*GPN K14A, D38A, GPN-AAA and D102A mutants (GTPase activity defect mutants) and **(B)** phosphorylation mutants (*Sa*GPN Y59D and Y59F), determined at 65 °C. Data represent mean +- s.d. of n=3 replicates. **(C)** Confirmation of plasmid-based expression of HA-tagged *Sa*GPN (GTPase activity defect mutants and phosphorylation mutants) in the Δ*saGPN* strain confirmed by western blot analysis. *S. acidocaldarius* cells were grown in nutrient-rich medium without uracil and cell samples were taken at OD_600_ of 0.4, which was analyzed by Western blot with α-HA antibody.

**
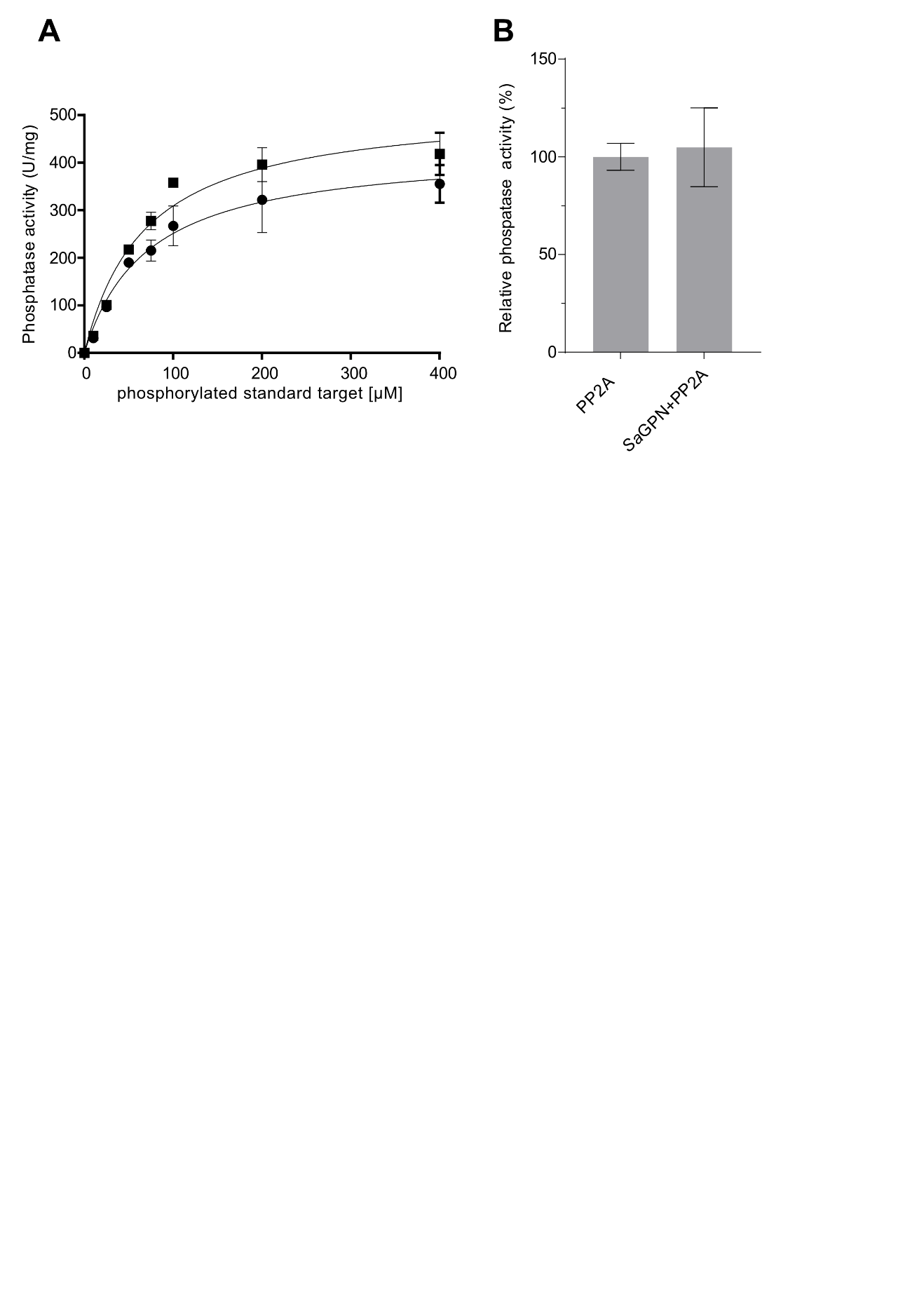
**

**Fig. S3. Phosphatase activity dependency of PP2A. (A)** Phosphatase activity of PP2A in absence (squares) or presence (circles) of *Sa*GPN. All experiments were performed in biological triplicates. **(B)** Phosphatase activity of PP2A in absence or presence of *Sa*GPN. All experiments were performed in biological triplicates. Data represent mean +- s.d. of n=3 replicates.

**
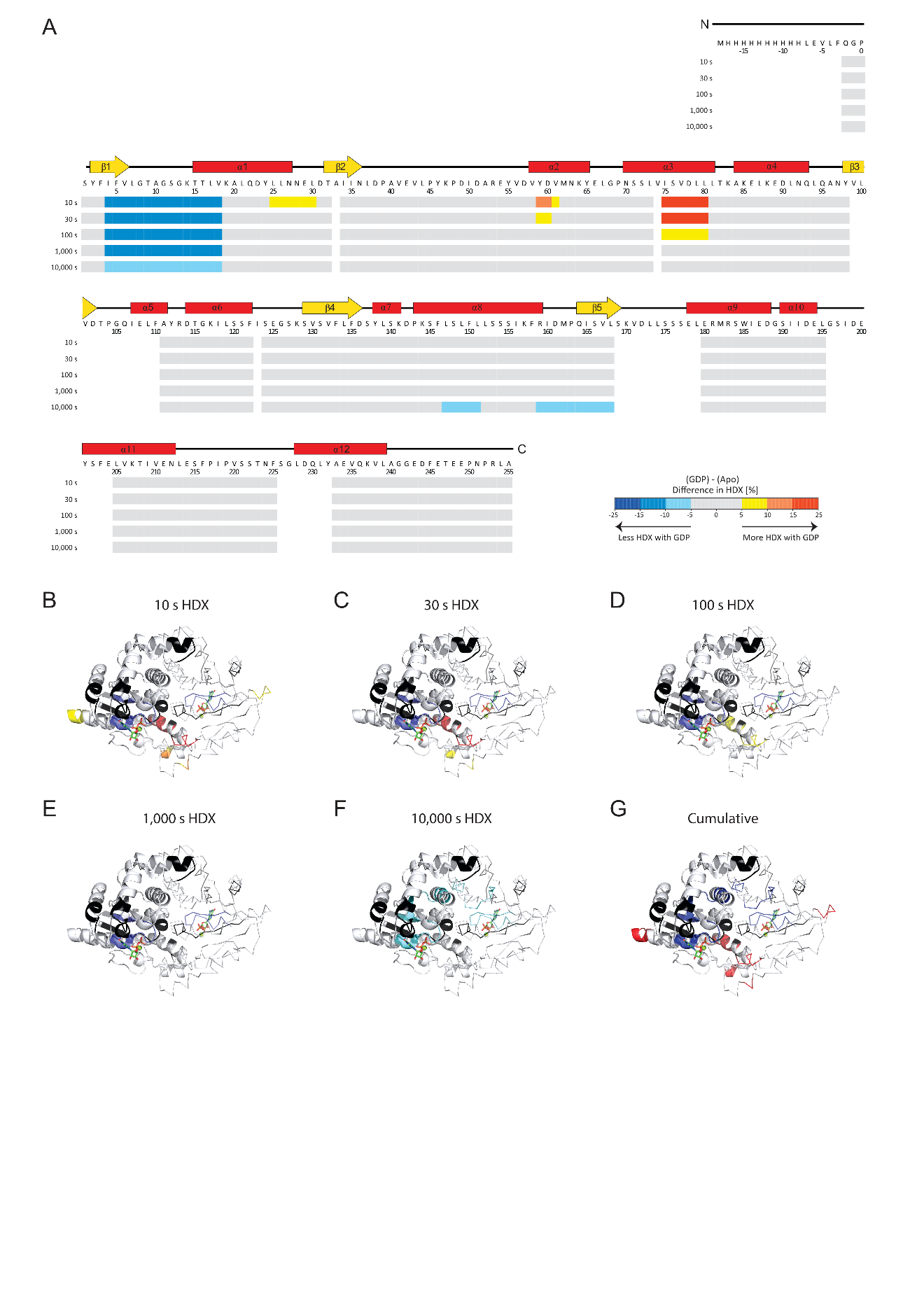
**

**Fig. S4. HDX difference of *Sa*GPN GDP state – Apo state.** **(A)** HDX of time points 10-10,000s mapped on sequence with secondary structure elements. Color code is explained by legend down right. **(B-F)** HDX of respective time points mapped on structure with one protomer in ribbon and one in cartoon view for orientation. **(G)** Cumulated HDX indicating if any HDX occurred during any time point with the highest magnitude in difference displayed on the structure.

**
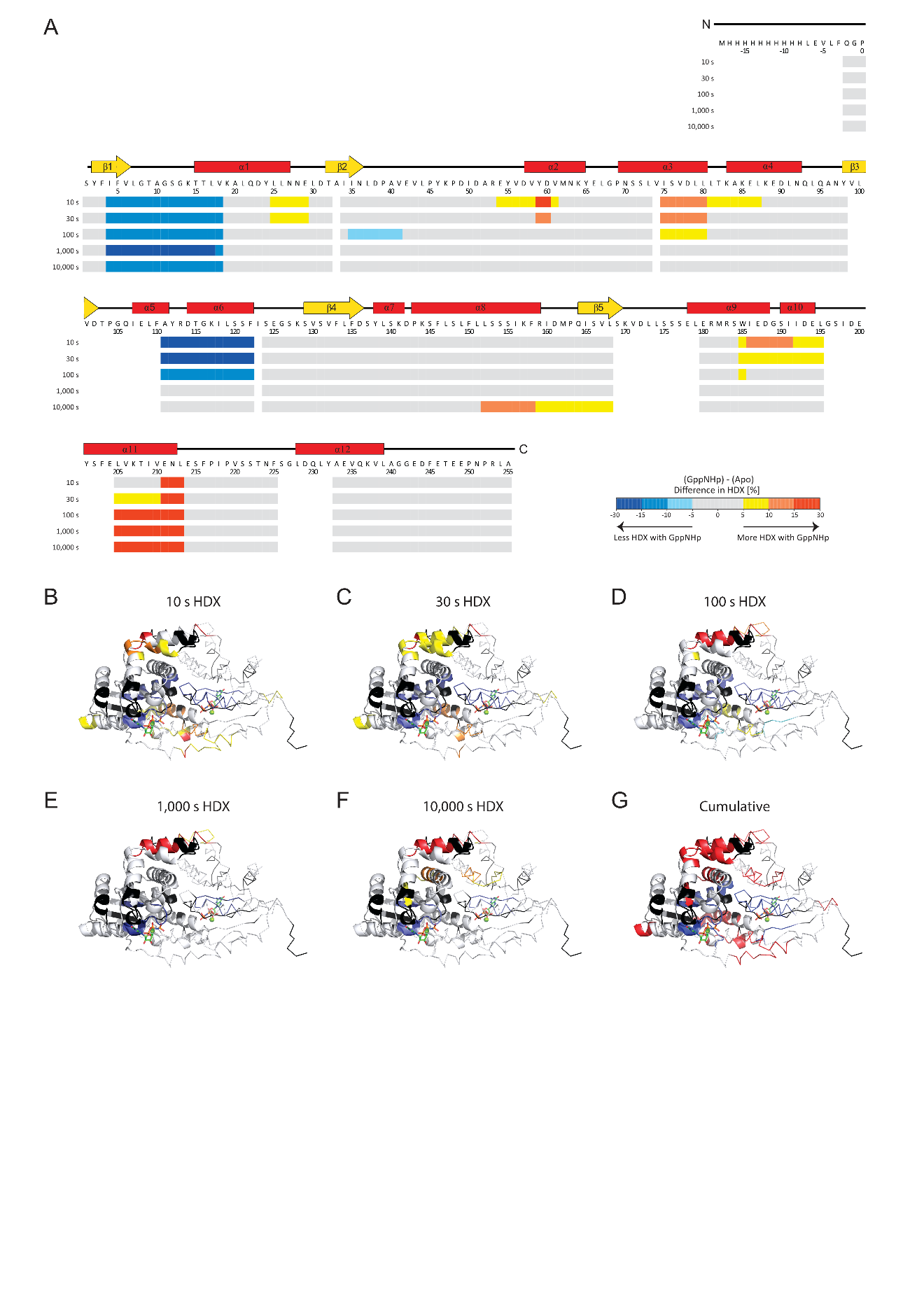
**

**Fig. S5. HDX difference of *Sa*GPN GppNHp state – Apo state. (A)** HDX of time points 10-10,000s mapped on sequence with secondary structure elements. Color code is explained by legend down right. **(B-F)** HDX of respective time points mapped on structure with one protomer in ribbon and one in cartoon view for orientation. **(G)** Cumulated HDX indicating if any HDX occurred during any time point with the highest magnitude in difference displayed on the structure.

**
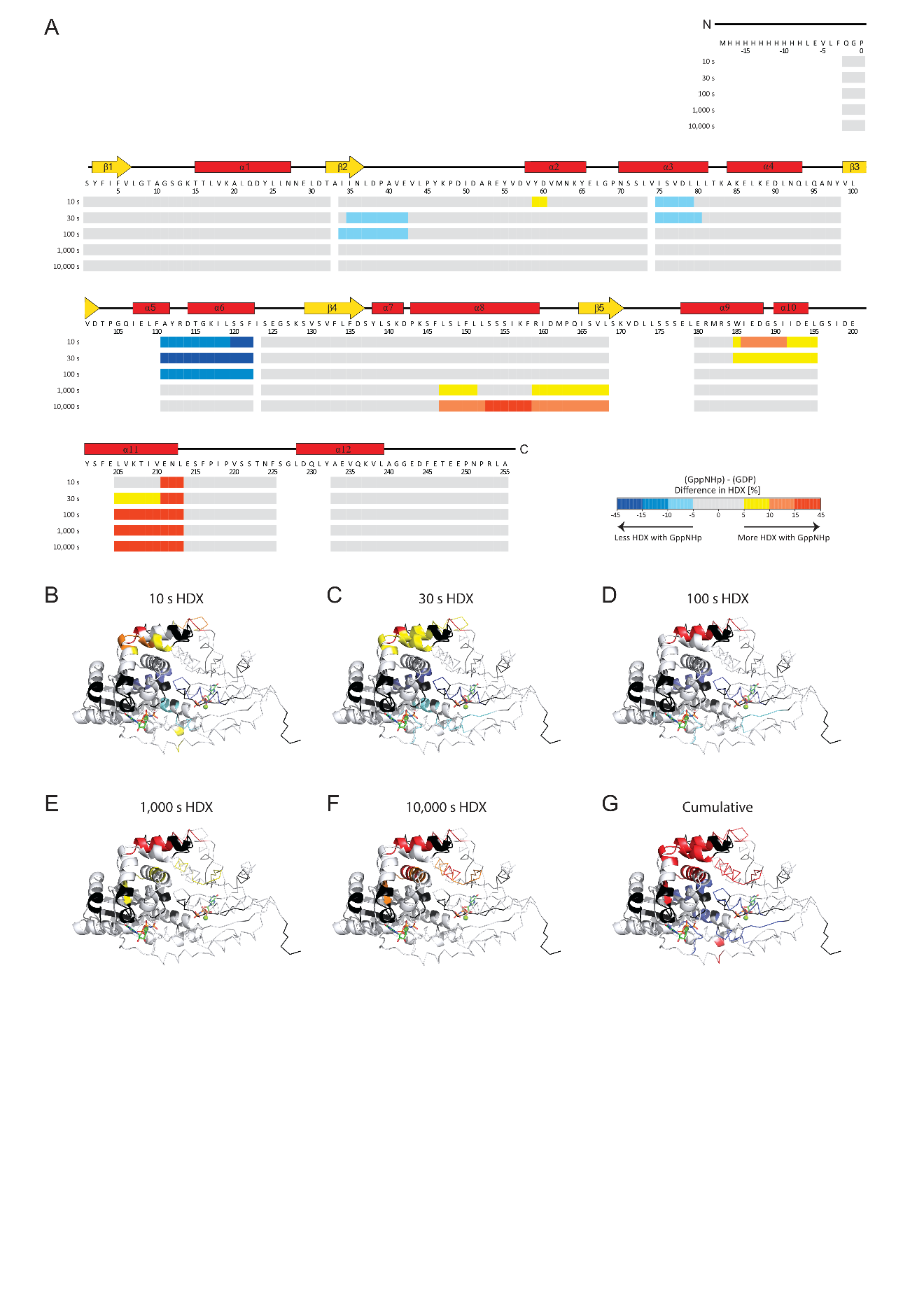
**

**Fig. S6. HDX difference of *Sa*GPN GppNHp state – GDP state. (A)** HDX of time points 10-10,000s mapped on sequence with secondary structure elements. Color code is explained by legend down right. **(B-F)** HDX of respective time points mapped on structure with one protomer in ribbon and one in cartoon view for orientation. **(G)** Cumulated HDX indicating if any HDX occurred during any time point with the highest magnitude in difference displayed on the structure.


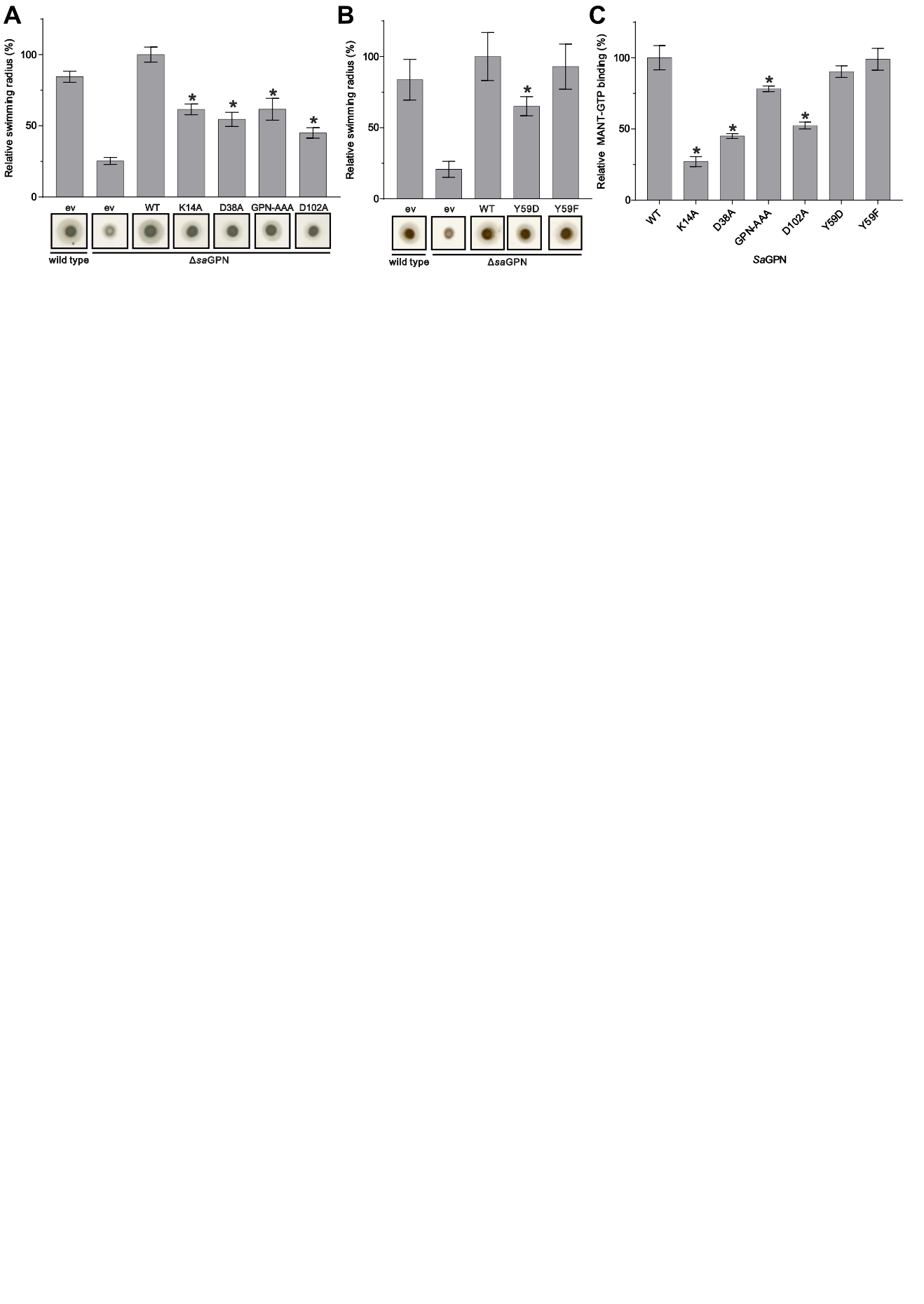


**Fig. S7. GTPase activity of *Sa*GPN is dispensable for motility of *S. acidocaldarius*. (A)** Δ*saGPN* strains were complemented with GTPase activity defect mutants varied in motifs G1 (K14A), G2 (D38A), GPN (AAA) and G3 (D102A), and their swimming motility was determined. ‘ev’ represents empty vector samples, i.e., ev Δ*saGPN* is the control sample. **(B)** Analysis of the potential phosphorylation target residue Y59 by complementation of Δ*saGPN* with non-phosphorylatable Y59F and non-homologous Y59D mutants. Swimming assays were carried out as in (A). Y59F could fully restore the swimming motility defect in contrast to Y59D, showing that phosphorylation at Y59 is not relevant for *Sa*GPNs biological role. Significant differences of swimming motility between complementation of Δ*saGPN* mutant with wild type *sa*GPN and with variants (p-value < 0.05) were indicated by an asterisk. **(C)** MANT-GTP binding assays of *Sa*GPN and its mutants show that GTP-binding is impaired by GTPase defect and Y59 mutants. Significant differences of MANT-GTP binding between wild type *Sa*GPN and variants (p-value < 0.05) were indicated by an asterisk. Data represent mean +- s.d. of n=3 replicates.

**
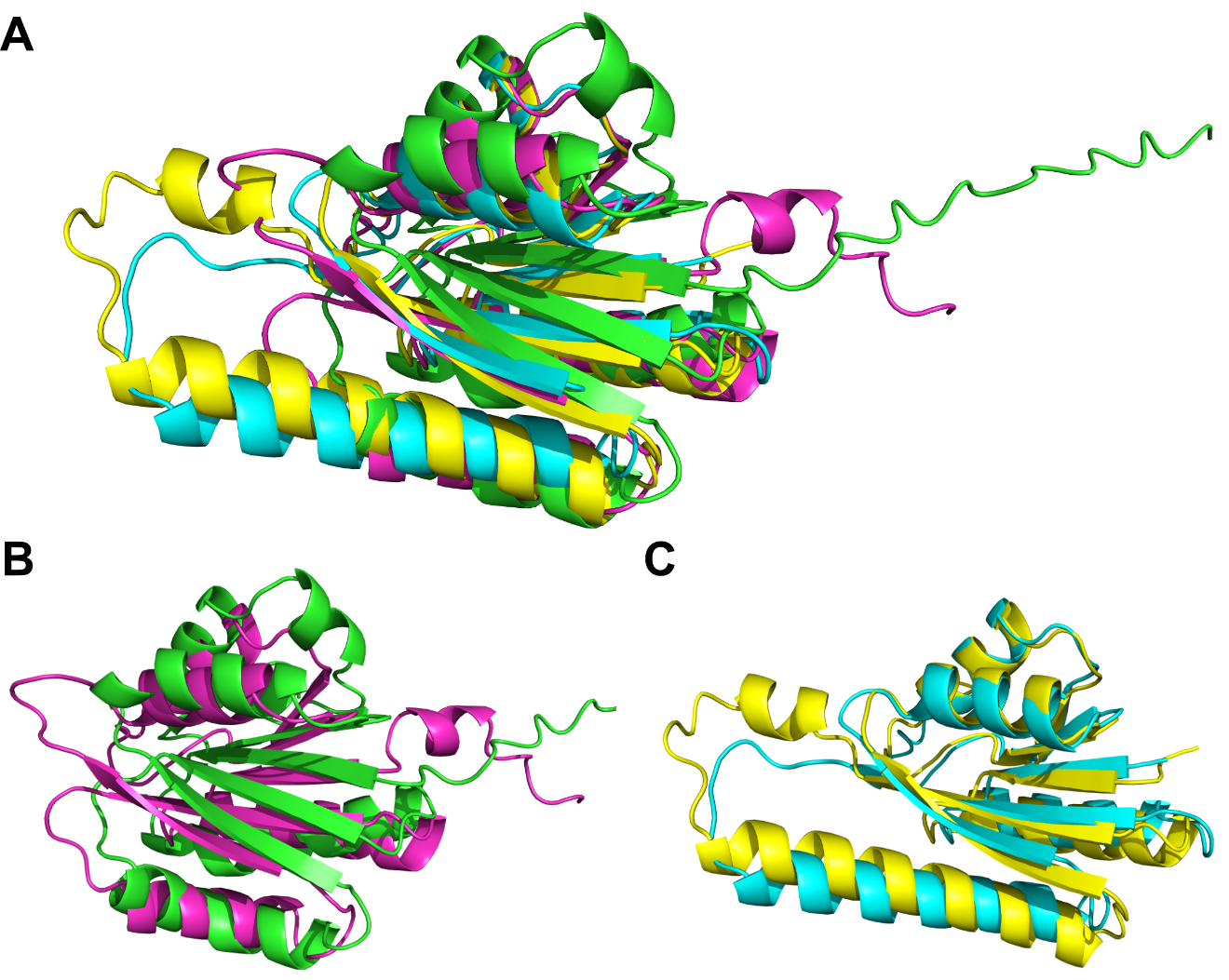
**

Fig. S8. Superposition of *S. acidocaldarius* Usp structure predictions. (A) Superposition of *Sa*UspA (cyan), *Sa*UspB (magenta), *Sa*UspC (yellow), and *Sa*UspD (green). AlphaFold models^1^ show good alignment of core Usp domains, despite low sequence identities between UspA, UspB and UspC (19-29%), and even lower of UspA/UspB/UspC to UspD (8-11%). (B) Superposition of *Sa*UspD and *Sa*UspB (seq. id. 11%, r.m.s.d. 3.504 Å for 64 Cα-positions) highlights their structural similarity. (C) Superposition of the pairwise related *Sa*UspA and *Sa*UspC (seq. id. 29%, r.m.s.d. 1.845 Å for 100 Cα-positions). AlphaFold models were created using google colab running AlphaFold v2.3.2. with standard settings.


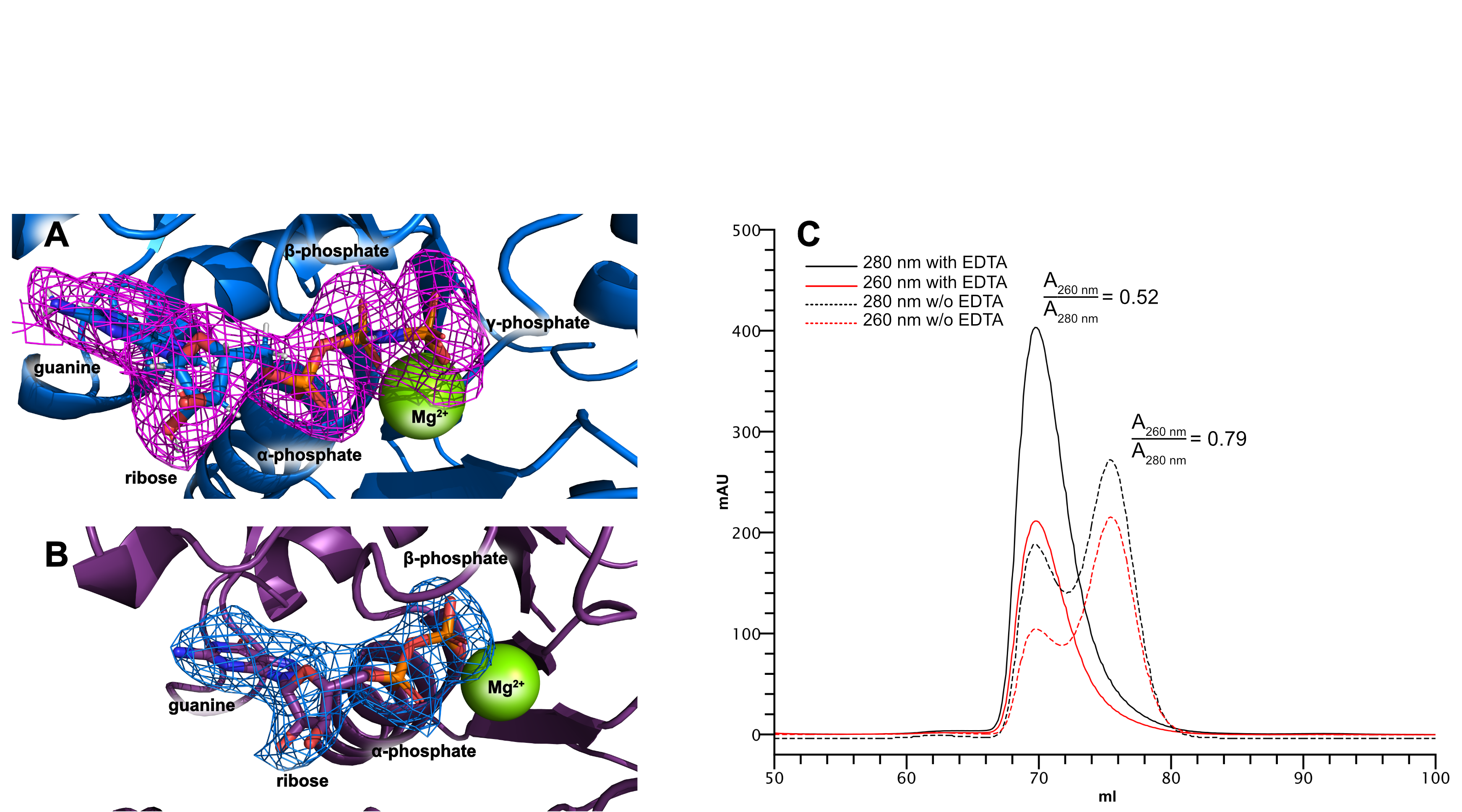


Fig. S9. F_o_-F_c_ omit maps displayed around ligands of *Sa*GPN structures confirming nucleotide states. (A) F_o_-F_c_ map around GppNHp reveals very well defined density including the γ-phosphate position. (B) F_o_-F_c_ map around GDP showing no excessive density for a potential γ-phosphate. (C) SEC chromatogram showing a representative *Sa*GPN purification with EDTA added during cell lysis and subsequent purification steps (continues lines) and without EDTA (dashed lines). Corresponding peak 260/280 nm ratios are given next to them. The 0.52 ratio peak refers to a nucleotide free extraction of *Sa*GPN while the 0.79 ratio refers to a nucleotide bound state. Black graphs display absorption at 280 nm while red graphs refer to absorption at 260 nm wavelength.

**
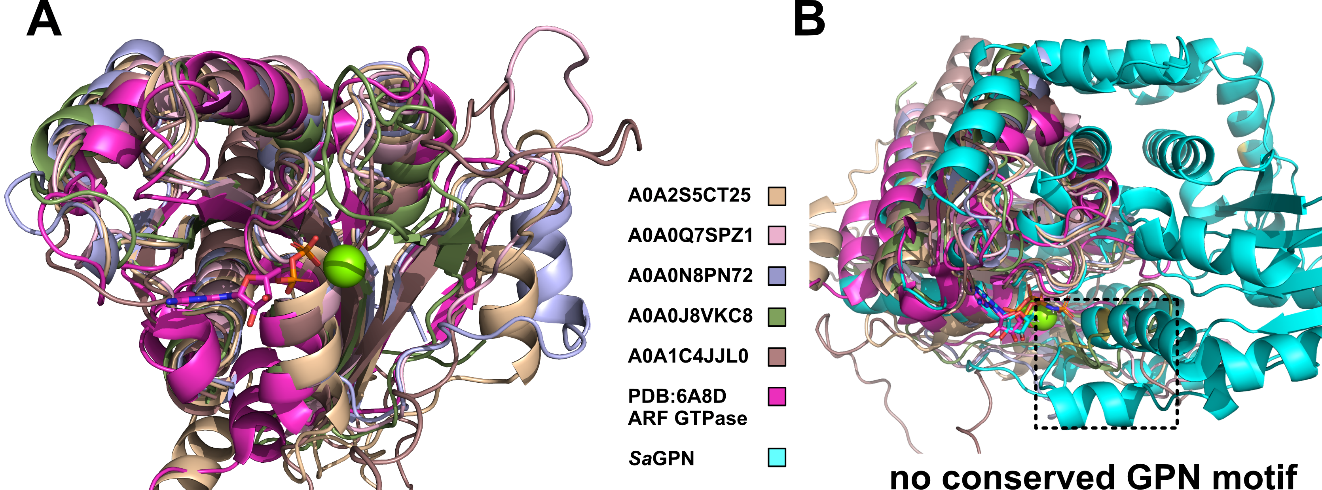
**

Fig. S10. Superposition of selected bacterial IPR004130 (GPN-loop GTPase) AlphaFold models superposed on *Chlamydomonas reinhardtii* ARF small GTPase. (A) Superposition of *C. reinhardtii* ARF small GTPase (PDB: 6A8D) revealing high similarity of bacterial annotated GPN-loop GTPase with ARF like small GTPases. (B) Annotated bacterial GPNs also lack the fundamental GPN-loop as visualized by the alignment with *Sa*GPN (PDB: 7ZHF). *C. reinhardtii* ARF small GTPase is colored in pink, *Sa*GPN in cyan and bacterial GPNs are colored in different pale colors.

**
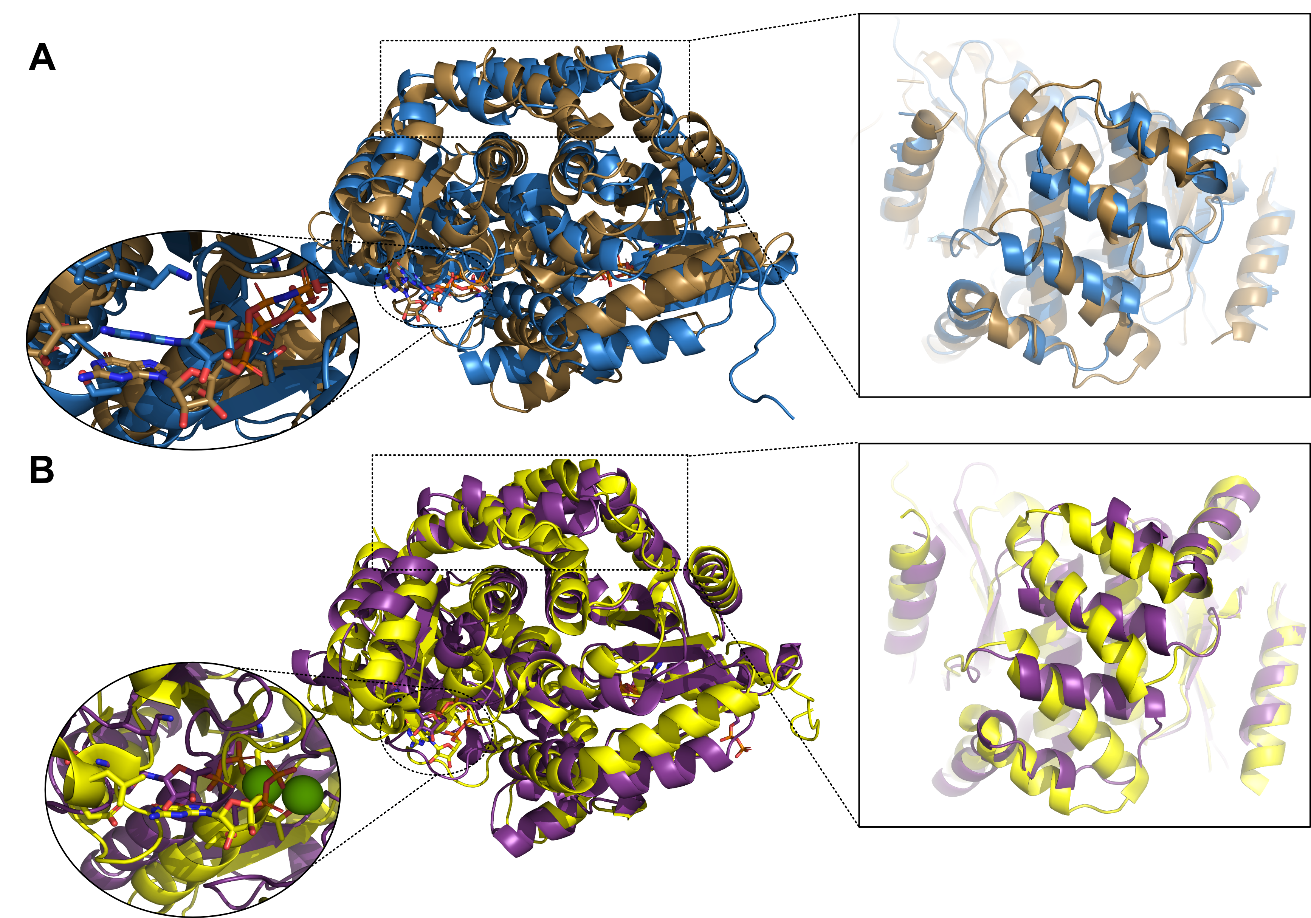
**

Fig. S11. Structural comparison of *Sa*GPN and *Pa*GPN in both states. (A) Structural comparison of SaGPN (GppNHp, blue) and *Pa*GPN (GTP, brown, PDB: 1YR8^2^) after superposition on monomer A. The roof helix region is highlighted as inlay in a top down view (right), the nucleotide cavity is shown as inlay (left). (B) Structural comparison of *Sa*GPN (GDP, purple) and *Pa*GPN (GDP, yellow, PDB: 1YRB^2^) focusing both the roof helix region in a top down view (right) and the nucleotide cavity (left).

**
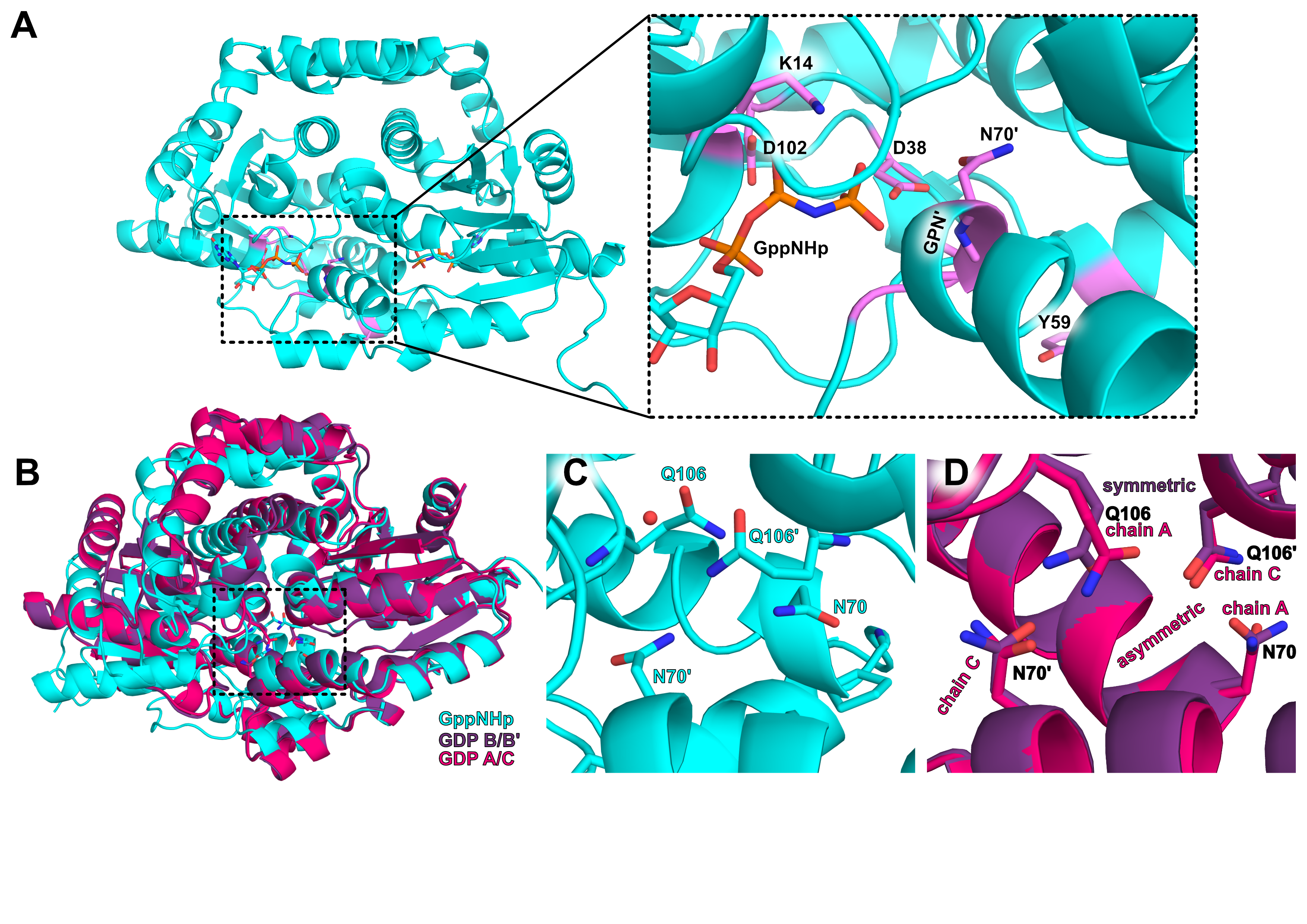
**

Fig. S12. Structural localization of SaGPN mutants and other structural highlights. (A) Highlighting of all residues that were mutated in this study for reference. (B) Structural comparison of different *Sa*GPN dimers (GDP chains A/C pink, GDP chains B/B’ purple, GppNHp cyan). (C) Focus on the dimer of *Sa*GPN GppNHp state showing the Q106/N70 interface. (D) Focus on the Q106/N70 interface of the two different *Sa*GPN·GDP dimers revealing the local asymmetry of the chain A/C dimer.

**Table S1.** Strains and plasmids in this study.

| **Strains/plasmids** | **Genotype** | **Source/Reference** |
| --- | --- | --- |
| ***Escherichia coli*** Top10 | F- *mcrA Δ(mrr-hsdRMS-mcrBC) φ80lacZΔM15 ΔlacX74 nupG recA1 araD139 Δ(ara-leu)7697 galE15 galK16 rpsL(Str^R^) endA1 λ^-^* | Invitrogen |
| ER1821 | λ^-^ F^-^ *glnX44 e14^-^(McrA^-^) rfbD1 endA1 thi-1 Δ(yjiT-opgB)114::IS10 (EcoKI R^-^ M^-^ McrBC^-^ Mrr^-^) + rpoS393(am) creC510 lrhA::IS3 ydeN::IS10* | New England Biolabs |
| Rosetta (DE3) pLysS | F^-^ *ompT hsdS*_B_(r_B_^-^ m_B_^-^) *gal dcm* (DE3) pLysSRARE (Cam^R^) | Novagen |
| ***Sulfolobus acidocaldarius*** |  |  |
| MW001 | *Sulfolobus acidocaldarius* DSM639 ∆*pyrE* | ^3^ |
| MW351 | MW001 Δ*saci1210* (Δ*arnA*) | ^4^ |
| MW332 | MW001 Δ*saci_1171Δsaci_1180 (ΔarnR ΔarnR1)* | ^5^ |
| MW829 | MW001 Δ*saci1281* (Δ*saGPN*) | This study |
| **Plasmids** |  |  |
| pSVA407 | Gene targeting plasmid, pGEM-T Easy backbone, *pyrEFSSO* and *lacSSSO* cassette; single crossover method | ^3^ |
| pSVA5142 | In-frame deletion of *saGPN*, cloned into pSVA407 with *Nco*I*, BamH*I | This study |
| pSVAaraFX-HA | pRN-1 based shuttle vector with *lacSSSO* reporter gene | ^6^ |
| pSVA5173 | Complementation of Δ*saGPN* with *saGPN* using its native promoter, cloned into pSVAaraFX-HA instead of *lacS* with *Sac*II and *Xho*I | This study |
| pSVA5174 | Complementation of Δ*saGPN* with *saGPN^K14A^* using its native promoter, cloned into pSVAaraFX-HA instead of *lacS* with *Sac*II and *Xho*I | This study |
| pSVA5175 | Complementation of Δ*saGPN* with *saGPN^D38A^* using its native promoter, cloned into pSVAaraFX-HA instead of *lacS* with *Sac*II and *Xho*I | This study |
| pSVA5176 | Complementation of Δ*saGPN* with *saGPN^GPN-AAA^* using its native promoter, cloned into pSVAaraFX-HA instead of *lacS* with *Sac*II and *Xho*I | This study |
| pSVA5177 | Complementation of Δ*saGPN* with *saGPN^D102A^* using its native promoter, cloned into pSVAaraFX-HA instead of *lacS* with *Sac*II and *Xho*I | This study |
| pSVA5193 | Complementation of Δ*saGPN* with *saGPN^Y59D^* using its native promoter, cloned into pSVAaraFX-HA instead of *lacS* with *Sac*II and *Xho*I | This study |
| pSVA13433 | Complementation of Δ*saGPN* with *saGPN^Y59F^* using its native promoter, cloned into pSVAaraFX-HA instead of *lacS* with *Sac*II and *Xho*I | This study |
| pSVA1037 | Saci0884 (*pp2a*) with C-terminal His-tag cloned into pETDuet-1 with *NcoI*, *BamHI* in MCSI | ^4^ |
| p7XC3H | FX cloning expression plasmid with C-term His tag | ^7^ |
| p7XNH3 | FX cloning expression plasmid with N-term His tag | ^7^ |
| p7XNS3 | FX cloning expression plasmid N-term Strep tag | ^8^ |
| pSVA5145 | *saGPN* cloned into p7XNS3 by FX cloning method | This study |
| pSVA5146 | *saGPN^K14A^* cloned into p7XNS3 by FX cloning method | This study |
| pSVA5147 | *saGPN^D38A^* cloned into p7XNS3 by FX cloning method | This study |
| pSVA5152 | *saGPN^GPN-AAA^* cloned into p7XC3H by FX cloning method | This study |
| pSVA5153 | *saGPN^D102A^* cloned into p7XC3H by FX cloning method | This study |
| pSVA5195 | *saGPN^Y59D^* cloned into p7XNH3 by FX cloning method | This study |
| pSVA13434 | *saGPN^Y59F^* cloned into p7XC3H by FX cloning method | This study |

**Table S2.** Primers used in this study.

| **Primer name** | **Sequence (5´- 3´)** | **Purpose** |
| --- | --- | --- |
|  | **primers for pSVA5142** |  |
| 9143 | AACTGCTCAAACCTAGGTCAGGATCCCTCGCAATATCCGGTATAG | Δ*saGPN* downstr rev |
| 9144 | ACGTCGCATGCTCCCGGCCGCCATGTCCCATATCGCCCATCACAG | Δ*saGPN* upstr fw |
| 9169 | GAACTTCGGCAGCCTTAACTAAAGTGGTCTTAC | Δ*saGPN* upstr rev ol |
| 9170 | AGTTAAGGCTGCCGAAGTTCAAAAGGTCTT | Δ*saGPN* downstr fw ol |
| 9108 | GCTCAAGACCGTACAAACTC | Δ*saGPN* check primer fw |
| 9109 | GTCACACCCAAGTTCTTGAG | Δ*saGPN* check primer rev |
|  | **primers for pSVA5173** |  |
| 9149 | GTCAGTGAGCGAGGAAGCCCACCGCCCTGCGGTGGATATAATGAC | *saGPN* compl fw |
| 9150 | CCGGAACGTCATACGGGTACTCGAGAAGCCTGGGGTTCGGTTCCT | *saGPN* compl rev |
|  | **primers for pSVA5145** |  |
| 9141 | ATATATGCTCTTCTAGTTACTTTATATTTGTACTAGGAACTGCA | *saGPN* expr fw |
| 9142 | TATATAGCTCTTCATGCAAGCCTGGGGTTCGGTTCCTCTGTTTC | *saGPN* expr rev |
|  | **Site-directed mutagenesis** |  |
| 9153 | TCAGGTGCAACCACTTTAGTTAAGGCTTTACAAGATTATTTG | *saGPN^K14A^* fw |
| 9154 | CCTTAACTAAAGTGGTTGCACCTGAACCTGCAGTTCCTAG | *saGPN^K14A^* rev |
| 9155 | TTAACCTAGCACCAGCAGTGGAAGTATTGCC | *SaGPN^D38A A^* fw |
| 9156 | CTGCTGGTGCTAGGTTAATTATTGCTGTGTCTAACTCATTG | *saGPN^D38A^* rev |
| 9157 | GAATTAGCAGCTGCATCCTCACTAGTGATTTCCGTAGATC | *saGPN^GPN-AAA^* fw |
| 9158 | TGAGGATGCAGCTGCTAATTCGTACTTATTCATCACATCATATAC | *saGPN^GPN-AAA^* rev |
| 9159 | CGTTTTAGTTGCAACTCCTGGTCAAATAGAACTTTTTGC | *saGPN^D102A^* fw |
| 9160 | CAGGAGTTGCAACTAAAACGTAATTGGCTTGAAGCTG | *saGPN^D102A^* rev |
| 11266 | GAGAGTACGTTGATGTAGATGATGTGATGAATAAGTACGA | *saGPN^Y59D^* fw |
| 11267 | ATCTACATCAACGTACTCTCTGGCATCG | *saGPN^Y59D^* rev |
| 11978 | GAGAGTACGTTGATGTATTTGATGTG | *saGPN^Y59F^* fw |
| 11979 | AAATACATCAACGTACTCTCTGGC | *saGPN^Y59F^* rev |
|  | **primers qRT-PCR** |  |
| 1480 | CCTGCAACATCTATCCATAACATACCGA | *secY*-qRT-PCR-fw |
| 1481 | CCTCATAGTGTATATGCTTTAGTAGTAG | *secY* -qRT-PCR-rev |
| 1424 | ACTGCGTCTACTGCGTTATCTTTATC | *flaB*-qRT-PCR-fw |
| 1425 | GGAGATAAGTCTACACTAGATACACCAGAA | *flaB*-qRT-PCR-rev |

**Table S3.** Crystallographic table for *Sa*GPN structures.

|  | *Sa*GPN•GppNHp (**7ZHF**) | *Sa*GPN•GDP (**7ZHK**)^†^ |
| --- | --- | --- |
| **Data collection and processing** | | |
| X-ray source, beamline | SLS, Beamline X06SA | SLS, Beamline X06SA |
| Detector | DECTRIS EIGER X 16M | DECTRIS EIGER X 16M |
| Wavelength (Å) | 1.000009 | 1.000031 |
| Space group | I 1 2 1 | C 2 2 2_1_ |
| Cell dimensions (*a*, *b*, *c*,  *α*, *β*, *γ*) | 46.30Å, 84.95Å, 75.32Å  90.00^°^, 95.62^°^, 90.00^°^ | 117.69Å, 141.88Å, 100.24Å  90.00^°^, 90.00^°^, 90.00^°^ |
| Resolution (Å) | 41.09 – 1.80 | 45.29 – 2.40 |
| Total reflections | 85,536 | 133,417 |
| Multiplicity | 3.5 | 4.3 |
| Unique reflections | 24,163 | 30,856 |
| Completeness (%)^#^ | 89.6 (64.1) | 76.8 (19.7) |
| *R*_merge_ | 0.048 (0.098) | 0.066 (1.22) |
| *CC1/2* | 0.999 (0.999) | 0.998 (0.325) |
| *I/σ(I)* | 9.1 (2.2) | 12.9 (1.5) |
| Wilson *B*-factor (Å^2^) | 41.2 | 52.8 |
| **Refinement** |  |  |
| Resolution (Å) | 29.5 – 1.80 (1.9-1.8) | 43.9 – 2.40 (2.5-2.4) |
| *R*_work_, *R*_free_ | 0.172, 0.204 (0.289) | 0.188 (0.256), 0.247 (0.332) |
| Reflections (working, test set) | 24,806 (2757), 796 (96) | 25,464 (716), 1283 (36) |
| r.m.s.d. from ideal: |  |  |
| Bond lengths (Å) | 0.006 | 0.002 |
| Bond angles (°) | 0.82 | 0.55 |
| Total number of atoms | 4,320 | 6,114 |
| Water | 196 | 351 |
| Chlorid Ions | 1 | 0 |
| Average *B*-factor (Å^2^) | 70.3 | 70.8 |
| Ligands | 55.6 | 51.4 |
| Ramachandran favored (%) | 98.8 | 99.6 |
| Ramachandran allowed (%) | 1.2 | 0.4 |
| Ramachandran outliers (%) | 0.0 | 0.0 |

Statistics for the highest-resolution shell are shown in parentheses.

^†^ Dataset was corrected by STARANISO^9^ for anisotropic diffraction. Cut-offs used direction 0.808 a* + 0.255 b* + 0.531 c* for best diffraction (2.40 Å), 0.023 a* + 0.993 b* + 0.115 c* for worst diffraction (3.25 Å). ^#^ Spherical completeness.

**Table S4 (separate file).** timsTOF data and GO term analysis of *S. acidocaldarius* WT and *saGPN* knockout strains.

**Table S5 (separate file).** *Sa*GPN HDX data and evaluation

Movie S1 (separate file). The movie shows a morph of the *Sa*GPNs•GppNHp state towards its GDP-bound state, visualizing the major allosteric changes upon nucleotide hydrolysis.

**SI References**

1. Jumper, J. *et al.* Highly accurate protein structure prediction with AlphaFold. *Nat. 2021 5967873* **596**, 583–589 (2021).

2. Gras, S. *et al.* Structural insights into a new homodimeric self-activated GTPase family. *EMBO Rep.* **8**, 569–575 (2007).

3. Wagner, M. *et al.* Versatile genetic tool box for the crenarchaeote Sulfolobus acidocaldarius. **3**, 1–12 (2012).

4. Reimann, J. *et al.* Regulation of archaella expression by the FHA and von Willebrand domain-containing proteins ArnA and ArnB in Sulfolobus acidocaldarius. *Mol. Microbiol.* **86**, 24–36 (2012).

5. Lassak, K., Peeters, E., Wróbel, S. & Albers, S. V. The one-component system ArnR: A membrane-bound activator of the crenarchaeal archaellum. *Mol. Microbiol.* **88**, (2013).

6. Van Der Kolk, N. *et al.* Identification of XylR, the activator of arabinose/xylose inducible regulon in Sulfolobus acidocaldarius and its application for homologous protein expression. *Front. Microbiol.* **11**, 1066 (2020).

7. Geertsma, E. R. & Dutzler, R. A versatile and efficient high-throughput cloning tool for structural biology. *Biochemistry* **50**, 3272–3278 (2011).

8. Ye, X. *et al.* The phosphatase PP2A interacts with ArnA and ArnB to regulate the oligomeric state and the stability of the ArnA/B complex. *Front. Microbiol.* **4**, 1849 (2020).

9. Vonrhein, C. *et al.* Advances in automated data analysis and processing within autoPROC , combined with improved characterisation, mitigation and visualisation of the anisotropy of diffraction limits using STARANISO . *Acta Crystallogr. Sect. A Found. Adv.* **74**, a360–a360 (2018).
